# Multiple poor sleep characteristics and metabolic abnormalities consistent with metabolic syndrome among white, black, and Hispanic/Latina women: modification by menopausal status

**DOI:** 10.1186/s13098-019-0413-2

**Published:** 2019-02-14

**Authors:** Symielle A. Gaston, Yong-Moon Park, Ketrell L. McWhorter, Dale P. Sandler, Chandra L. Jackson

**Affiliations:** 1 Epidemiology Branch, Department of Health and Human Services, National Institute of Environmental Health Sciences, National Institutes of Health, Research Triangle Park, NC USA; 2 Intramural Program, Department of Health and Human Services, National Institute of Minority Health and Health Disparities, National Institutes of Health, Bethesda, MD USA

**Keywords:** Metabolic syndrome, Sleep, African Americans, Hispanic Americans, Whites, Women, Menopause

## Abstract

**Background:**

Poor sleep is a potential risk factor for metabolic syndrome (MetS), and its relationship with MetS may vary by race/ethnicity and menopausal status among women.

**Methods:**

We used Sister Study enrollment data from 2003 to 2009 to investigate the cross-sectional associations between multiple subjective sleep characteristics and having ≥ 3 prevalent metabolic abnormalities consistent with MetS among white, black, and Hispanic/Latina women. Self-reported sleep characteristics included average sleep duration (short [< 7 h] vs. recommended [7–9 h]), sleep debt (≥ 2-h difference between shortest and longest sleep duration, napping ≥ 3 times/week, and insomnia symptoms (difficulty falling or staying asleep). We used Poisson regression with robust variance to estimate adjusted prevalence ratios (PRs) and 95% confidence intervals (CIs) to compare MetS prevalence between women with poor sleep (e.g., short sleep, sleep debt, frequent napping, or insomnia symptoms [all yes vs. no]) and non-poor sleep within menopausal status categories (premenopausal or postmenopausal). We adjusted for sociodemographic characteristics, mental health, and health behaviors.

**Results:**

Among 38,007 eligible women (13,988 premenopausal, 24,019 postmenopausal), mean age was 55 ± 8.8 years, racial/ethnic composition was 86.63% white, 8.53% black, and 4.84% Hispanic/Latina, and 12% had MetS. Associations between certain poor sleep characteristics [i.e., short sleep (PR_premenopausal_ = 1.23 [95% CI 1.06–1.42], PR_postmenopausal_ = 1.09 [1.02–1.16], p_short sleep*menopause_ = 0.0070) and insomnia symptoms (PR_premenopausal_ = 1.21 [1.05–1.41], PR_postmenopausal_ = 1.11 [1.05–1.18], p_insomnia symptoms*menopause_ = 0.035)] and prevalent MetS were stronger among premenopausal compared to postmenopausal women, but did not vary by race/ethnicity. Associations between concurrent short sleep/insomnia symptoms and MetS were stronger among white and Hispanic/Latina postmenopausal women compared to their black counterparts. Menopausal status and race/ethnicity did not modify positive associations for other poor sleep characteristics.

**Conclusions:**

Poor sleep was positively associated with MetS prevalence. Associations between individual poor sleep characteristics (i.e., short sleep, insomnia symptoms) were stronger among premenopausal compared to postmenopausal women but did not vary by race/ethnicity.

**Electronic supplementary material:**

The online version of this article (10.1186/s13098-019-0413-2) contains supplementary material, which is available to authorized users.

## Background

Metabolic syndrome (MetS) is a cluster of cardiometabolic risk factors [e.g., elevated blood pressure (BP), elevated fasting blood glucose, dyslipidemia, abdominal obesity] that is associated with a twofold higher risk of cardiovascular disease as well as a 50% higher risk of all-cause mortality [1, 2]. In the United States (US), the prevalence of MetS appears to have increased over the past two decades with a recent estimate of 34% among adults (33% [men] and 35% [women]) [3, 4]. Among women, MetS prevalence for non-Hispanic white and non-Hispanic black (hereafter referred to as white and black) women, increased by 44% and 41% from 1988 to 2012 [3]. Although MetS prevalence increased by only 2% among Mexican women, black and Mexican women were 20% more likely than white women to have MetS in this same period [3]. Primary and secondary prevention of MetS is warranted, especially among women, given the associated health sequelae, increasing prevalence, and observed racial/ethnic disparities.

Meta-analyses suggest that poor sleep duration is a potential risk factor for MetS [5–7]; however, observational studies measuring sleep quality have had mixed results [8–17]. Nonetheless, experimental studies support associations between short-term sleep deprivation and physiological changes related to individual MetS components like weight gain, insulin resistance, and elevated nocturnal BP [18–20]. Overall, one-third of Americans do not get the 7–9 h of quality, uninterrupted sleep recommended by the National Sleep Foundation [21, 22]. Furthermore, blacks and Hispanics/Latinos are two-to-four times more likely to report inadequate sleep compared to whites, and racial/ethnic disparities in sleep are likely contributors to racial/ethnic disparities in poor cardiometabolic health outcomes like frequently observed higher obesity prevalence among these racial/ethnic groups [3, 4, 23–26].

Among women, menopausal status may affect relationships between sleep characteristics and MetS. Postmenopausal women have been shown to have shorter sleep duration, worse sleep quality, and increased MetS risk compared to premenopausal women, independent of age (e.g., among younger women with surgical menopause) and likely due to declines in sex hormones (e.g., estrogen) [27–30]. Although poor sleep and higher MetS risk may be due to menopause-related physiologic changes among menopausal women, poor sleep may act more as a risk factor for MetS among premenopausal women. Despite commonly-observed racial/ethnic and menopausal status differences in sleep and cardiometabolic health, few studies have investigated these characteristics as modifiers of sleep-MetS relationships [3, 4, 23, 24, 27–29, 31–33]. To address this important gap, the objective of our study was to investigate the relationship between multiple sleep characteristics and prevalent metabolic abnormalities consistent with MetS among a multi-ethnic cohort of US women, and to determine whether these relationships vary by menopausal status and race/ethnicity.

## Methods

### The Sister Study Cohort

In 2018, we analyzed baseline data from data release 5.0.2 of the Sister Study, a prospective cohort study of 50,884 US women designed to investigate environmental and genetic risk factors for breast cancer among women aged 35–74 years. To be eligible for the study, participants had to live in the US, meet age criteria, have a sister diagnosed with breast cancer, and be without breast cancer. Methods and recruitment strategies are described in detail elsewhere [34]. Briefly, women were recruited from the 50 US states and Puerto Rico. Recruitment strategies included word-of-mouth and flyer distribution at breast cancer support and advocacy groups and events; outreach at hospitals, medical centers, and other institutions the target population would visit; direct mailings; and mass media. Special recruitment efforts were made to ensure inclusion of typically underrepresented women including non-whites, older women, and women of lower socioeconomic status. Baseline data collection occurred from 2003 to 2009 and included self-administered questionnaires, a two-part computer-assisted telephone interview, and a home visit that included anthropometric measurements, biologic specimen collection, and home environmental samples. The National Institute of Environmental Health Sciences Institutional Review Board and the Copernicus Group Independent Review Board approved the Sister Study protocol, and all participants provided informed consent.

### Study participants

Women were excluded in a stepwise manner if they self-identified as any race/ethnicity other than white, black, or Hispanic/Latina (e.g., non-Hispanic Asian) due to low sample size (n = 1334); were currently pregnant, breastfeeding, or with pre-existing medical conditions (i.e., cancer, diabetes diagnosis before age 20 years or between ages 20 and 34 years along with continuous insulin usage, cardiovascular/cerebrovascular disease, n = 5529); were current shift workers (n = 288); or were missing values or had unknown timing of excluded medical conditions (n = 1230). Of the eligible participants, we excluded from analyses those with missing or implausible values for MetS components or potential confounders (n = 4147) or who self-reported long (> 9 h) sleep duration (due to small sample size, n = 349) (Additional file 1: Figure S1).

### Measures

#### Sleep characteristics

Detailed questionnaires used to ascertain sleep characteristics are publicly available at https://sisterstudy.niehs.nih.gov/English/enroll-data.htm. Participants reported hours and minutes of habitual sleep duration by responding to ‘About how many hours and/or minutes of sleep per (night/day) do you get on average?’. We rounded responses to the nearest tenth of an hour and applied National Sleep Foundation recommendations, categorizing average sleep duration as *short* (< 7.0 h) and *recommended* (7.0–9.0 h) [22]. We determined *inconsistent weekly sleep patterns* (yes vs. no) based on whether participants reported consistent (could vary day-by-day but were stable from week-to-week) or inconsistent wake-up times and bedtimes during the prior 6 weeks. Participants with *consistent weekly sleep patterns* reported daily bedtimes and wake-up times from which we directly calculated longest and shortest sleep duration. Participants with *inconsistent weekly sleep patterns* reported their longest and shortest average sleep duration. Among all participants, we defined *sleep debt* as a ≥ 2-h vs. < 2-h difference between longest and shortest sleep duration. Participants reported napping frequency, and we dichotomized *frequent napping* as ≥ 3 naps per week vs. < 3 naps per week. Participants also reported the average time taken to fall asleep and frequency of night awakenings. *Insomnia symptoms* included either *difficulty falling asleep* (taking ≥ 30 min vs. < 30 min to fall asleep on average) or *difficulty staying asleep* (awakening ≥ 3 times per night/day, ≥ 3 nights/days per week vs. awakening < 3 times per night/day and/or < 3 nights/days per week) vs. neither, which were also individually assessed. We examined the combination of *short sleep duration* and *insomnia symptoms*. Lastly, we calculated a cumulative sleep score which was the sum of yes responses to the main sleep characteristics (*short sleep duration*, *inconsistent weekly sleep patterns*, *sleep debt*, *frequent napping*, and *insomnia symptoms* [range: 0–5]).

#### Prevalent metabolic abnormalities consistent with metabolic syndrome

To be classified as having prevalent metabolic abnormalities consistent with MetS (hereafter, referred to as MetS), participants had to meet at least three criteria related to hypertension, abdominal obesity, dyslipidemia, and prediabetes/type 2 diabetes mellitus (T2DM). We adapted the harmonized definition for MetS as outlined by Alberti et al. [35] by using self-reported measures for dyslipidemia and prediabetes/T2DM. During the baseline examination, technicians measured waist circumference (WC) and took three BP measurements, if possible. Participants met criteria for abdominal obesity if their measured WC averaged ≥ 88 cm. BP measurements were taken between 1 and 2 min apart, and we averaged the latter two BP measurements. If either of the latter two measurements was unavailable, we included the first BP measurement (3.8%). We classified participants with a systolic BP ≥ 130 mmHg, a diastolic BP ≥ 85 mmHg, or report of a physician’s diagnosis of hypertension and antihypertensive medication use in the past 12 months as having hypertension. We considered participants as having dyslipidemia if they self-reported a physician or healthcare provider diagnosis of high cholesterol, high triglycerides, or current use (within the past 12 months) of medications to lower triglycerides or raise high density lipoprotein cholesterol (i.e., fibrates, niacin, long-chain omega-3 fatty acids, statins). T2DM was defined as self-report of physician-diagnosed diabetes or diabetes medication (including insulin) usage in the past 12 months. Prediabetes was based on self-reported physician-diagnosed borderline diabetes.

#### Covariates

We considered self-reported (unless otherwise stated) potential confounders including sociodemographic, health behavior, and clinical characteristics [3, 8, 14]. Sociodemographic factors included age (in years), educational attainment (≤ high school, associate’s/technical degree or some college, ≥ college graduate), current employment (yes vs. no), annual household income (< $20,000, $20,000–$49,999, $50,000–$99,999, ≥ $100,000), household size, whether children (aged ≤ 18 years) were in the household (yes vs. no), and marital status (married/living as married, divorced/separated/widowed, never married). Health behaviors included smoking status (current, former, never), alcohol consumption (never/former [0 drinks/week], light/moderate [1 to ≤ 7 drinks/week], heavy [> 7 drinks/week]), the sum of average total metabolic equivalent (METs) hours per week of self-reported ‘leisure-time, occupational, and daily activities’ physical activity in the past 12 months calculated using the Compendium of Physical Activities [36, 37], diet quality (Healthy Eating Index [HEI] score [38] and daily glycemic load [glucose scale] [39, 40]) calculated from responses to a modified Block 1998 Food Frequency Questionnaire, and reported sleep medication use to fall or stay asleep in the past 6 weeks (yes vs. no). Clinical characteristics included body mass index (BMI) calculated from objectively-measured height and weight (kg/m^2^) and categorized as underweight (< 18.5 kg/m^2^), normal weight (18.5–24.9 kg/m^2^), overweight (25.0–29.9 kg/m^2^), obese (≥ 30.0 kg/m^2^) [41]; reported physician-diagnosis of clinical depression or bipolar disorder (yes vs. no); and hormone replacement therapy use (yes vs. no).

#### Potential modifiers: menopausal status and race/ethnicity

We considered menopausal status and race/ethnicity as potential moderators of relationships between poor sleep and an MetS based on prior literature [3, 26, 27, 29, 42]. Self-reported menopausal status was dichotomized as premenopausal and postmenopausal (whether natural or non-natural [i.e., surgical or due to treatments] that caused cessation of menstruation). We categorized participants as white, black, or Hispanic/Latina if they reported only non-Hispanic white race/ethnicity, only non-Hispanic black race/ethnicity, or Hispanic/Latina ethnicity and any race, respectively. We used white as the reference category because whites represented the largest sample size and had the highest likelihood of both non-poor sleep and lowest prevalence of MetS [3, 26].

### Statistical analysis

We used Poisson regression with robust variance estimation [43] to calculate adjusted prevalence ratios (PRs) and 95% confidence intervals (CIs) to compare MetS prevalence between women with poor sleep to women with recommended sleep (yes vs. no for short sleep, inconsistent weekly sleep patterns, sleep debt, frequent napping, insomnia symptoms, difficulty falling asleep, or difficulty staying asleep) for premenopausal and postmenopausal women, separately. A two-sided p-value of 0.05 was used to determine statistical significance in all models. Based on directed acyclic graphs [44] and the prior literature, all adjusted models included age, educational attainment, annual household income, smoking status, alcohol consumption, healthy eating index score, log-transformed metabolic equivalents, hormone replacement therapy use, clinical depression/bipolar disorder, and sleep medication use. Models for sleep debt were additionally adjusted for consistent weekly sleep patterns. Models including all participants were additionally adjusted for race/ethnicity. We determined statistical significance for a menopausal status-by-sleep characteristic interaction term in models that included all participants. Within each menopausal status category, we also included a sleep parameter-by-race/ethnicity interaction term and then stratified models by race/ethnicity. All analyses were performed using SAS software, version 9.4 of the SAS System for Windows (Cary, NC).

### Sensitivity analysis

We performed six independent sensitivity analyses. First, we adjusted for use of medication for dyslipidemia because of the potential for adverse sleep-related side effects related to medication use. Second, among menopausal women, we stratified by natural menopause (yes vs. no) due to the higher risk of MetS associated with surgical compared to natural menopause [29]. Third, we recategorized participants with diastolic BP ≥ 80 mmHg as hypertensive based on recent recommendations of the American College of Cardiology/American Heart Association Task Force and reran adjusted models [45]. Fourth, we additionally stratified the analytic sample by sleep medication use. Fifth, we mutually adjusted for all individual sleep characteristics to determine which associations were robust after adjustment for other sleep characteristics. Lastly, to determine whether associations between poor sleep and individual MetS components varied by subgroup, we investigated individual metabolic abnormalities as outcomes.

## Results

### Study population

Our analytic sample consisted of 38,007 participants (Additional file 1: Figure S1). Mean age was 55 ± 8.8 years and racial/ethnic composition was 86.6% white, 8.5% black, and 4.8% Hispanic/Latina (Table 1). Black women were the most likely to have at least a bachelor’s degree and be currently employed compared to white and Hispanic/Latina women. Blacks and Hispanics/Latinas had lower annual household income, on average, compared to whites. Most of the participants were postmenopausal (63%). Overall, postmenopausal women were generally more likely to have poor sleep characteristics compared to premenopausal women. The most notable racial/ethnic differences in sleep characteristics were for sleep duration: black women had the highest prevalence of *short sleep duration* (53% premenopausal and 53% postmenopausal for blacks vs. 24% premenopausal and 26% postmenopausal for whites and 33% premenopausal and 41% postmenopausal for Hispanics/Latinas). Among both premenopausal and postmenopausal participants, black and Hispanic/Latina women had similarly higher prevalence of *inconsistent weekly sleep patterns*, *sleep debt*, *frequent napping*, *insomnia symptoms,* and both *short sleep and insomnia symptoms* compared to white women. Compared to premenopausal women, postmenopausal women also had higher prevalence of MetS (white—14% vs. 4.6%, black—28% vs. 12%, and Hispanic/Latina—21% vs. 8.7%).

**Table 1:** Sociodemographic, health behavior, and clinical characteristics of eligible Sister Study participants (N = 38,007)

	Total N = 38,007			White n = 32,925 (86.63%)			Black n = 3243 (8.53%)			Hispanic/Latina n = 1839 (4.84%)		
	All	Menopausal status		All	Menopausal status		All	Menopausal status		All	Menopausal status	
		Pre- n = 13,988 36.8%	Post- n = 24,019 63.2%		Pre- n = 11,757 35.7%	Post- n = 21,168 64.3%		Pre- n = 1417 43.4%	Post- n = 1826 56.3%		Pre- n = 814 44.3%	Post- n = 1025 55.7%
*Sociodemographic characteristics*												
Mean age ± SD (years)	55.0 ± 8.77	46.8 ± 5.12	59.8 ± 6.62	55.3 ± 8.79	46.8 ± 5.10	60.1 ± 6.61	53.1 ± 8.14	46.7 ± 5.16	58.1 ± 6.33	52.7 ± 8.88	45.6 ± 5.25	58.3 ± 6.89
Educational attainment												
≤ High school	14.5	11.8	16.1	14.6	11.9	16.1	9.56	7.83	10.9	22.0	17.2	25.8
Some college or technical degree	32.9	31.9	33.5	32.7	31.5	33.3	34.0	34.2	33.8	35.0	34.4	35.5
≥ College (bachelor’s or greater)	52.6	56.3	50.5	52.8	56.7	50.6	56.5	58.0	55.3	43.0	48.4	38.7
Currently employed (yes)	68.4	83.4	59.6	67.7	83.0	59.2	77.0	89.6	67.3	64.4	78.6	53.2
Annual household income												
Less than $20,000	3.77	2.48	4.51	2.86	1.60	3.57	4.93	4.38	5.37	17.8	11.9	22.5
$20,000 to $49,999	19.8	14.2	23.1	18.8	12.4	22.4	24.1	21.7	25.9	30.2	27.3	32.6
$50,000 to $99,999	41.5	41.9	41.3	41.8	42.4	41.5	44.3	42.7	45.5	31.4	34.2	29.3
$100,000 or more	34.9	41.4	31.1	36.5	43.6	32.6	26.7	31.2	23.2	20.5	26.7	15.6
Household size, median (IQR)	2 (2–3)	3 (2–4)	2 (2–2)	2 (2–3)	3 (2–4)	2 (2–2)	2 (1–3)	3 (2–4)	2 (1–2)	2 (2–4)	3 (2–4)	2 (2–3)
At least one child aged < 18 years in household (yes)	27.7	55.2	11.7	26.6	55.4	10.5	33.5	51.2	19.8	37.5	59.1	20.3
Marital status												
Married/living as married	75.5	79.6	73.1	78.1	82.6	75.6	53.6	57.1	51.0	68.2	76.2	61.9
Divorced/separated/widowed	19.0	13.1	22.5	17.4	11.6	20.6	32.4	24.1	38.9	24.7	15.6	31.9
Single/never married	5.47	7.31	4.40	4.55	5.86	3.82	14.0	18.8	10.2	7.12	8.23	6.24
*Health behaviors*												
Smoking status												
Current	7.67	8.47	7.20	7.59	8.54	7.06	9.28	8.47	9.91	6.25	7.49	5.27
Former	35.0	23.4	38.8	36.6	30.3	40.2	25.2	17.5	31.1	22.9	20.0	25.2
Never	57.3	63.2	54.0	55.8	61.2	52.8	65.6	74.0	60.0	70.9	72.5	69.6
Alcohol consumption (past 12 months)												
Heavy (> 7 drinks/week)	11.3	11.2	11.5	12.3	12.3	12.4	4.69	4.73	4.65	5.22	6.02	4.59
Light/moderate (≤ 7 drinks/week)	71.2	74.5	69.3	71.9	75.3	70.0	66.7	70.3	63.9	66.4	70.5	63.1
Nondrinker (never/former)	17.5	14.3	19.3	15.8	12.4	17.6	28.7	25.0	31.5	28.4	23.5	32.3
METs-hours/week ± SD	50.9 ± 31.1	50.3 ± 31.1	51.3 ± 31.2	51.5 ± 31.2	51.0 ± 31.3	51.8 ± 31.2	45.4 ± 29.6	45.3 ± 29.7	45.4 ± 29.5	49.7 ± 30.8	48.2 ± 29.6	51.0 ± 31.8
Healthy Eating Index score ± SD	62.3 ± 12.2	61.3 ± 12.0	62.8 ± 12.2	62.8 ± 12.2	61.8 ± 12.1	63.3 ± 12.2	58.6 ± 11.3	58.2 ± 10.9	58.9 ± 11.5	60.1 ± 11.8	59.8 ± 11.4	60.3 ± 12.2
Daily glycemic load, glucose scale ± SD	85.0 ± 39.1	87.8 ± 40.7	83.3 ± 38.1	83.8 ± 36.8	86.4 ± 38.5	82.4 ± 35.7	93.3 ± 52.8	96.9 ± 52.6	90.5 ± 52.8	91.0 ± 47.8	92.1 ± 45.4	90.1 ± 49.7
Habitual sleep duration category^A^												
Short sleep duration (< 7 h)	28.3	27.5	28.7	25.3	24.1	26.0	53.1	52.7	53.3	37.4	33.3	40.6
Recommended sleep duration (7–9 h)	71.7	72.5	71.3	74.7	75.9	74.0	46.9	47.3	46.7	62.6	66.8	59.4
Inconsistent weekly sleep patterns (yes)	14.2	11.6	15.8	13.2	10.4	14.8	21.1	18.6	23.1	20.0	16.4	22.8
Sleep debt (yes)	25.1	28.5	23.2	23.8	27.0	22.0	34.1	37.2	31.7	33.2	34.9	31.7
Napping ≥ 3 times/week (yes)	9.58	7.22	11.0	9.04	6.64	10.4	13.3	10.7	15.3	12.7	9.71	15.1
Insomnia symptoms (yes)	26.0	21.8	28.4	24.7	20.3	27.2	33.3	30.3	35.6	35.4	29.8	39.8
Difficulty falling asleep (yes)	17.1	14.6	18.5	15.4	12.8	16.9	27.4	25.1	29.3	28.4	23.1	32.5
Difficulty staying asleep (yes)	13.1	10.3	14.8	13.2	10.3	14.8	11.5	9.33	13.1	14.5	12.0	16.6
Short sleep duration and insomnia symptoms (yes)	11.4	9.78	12.3	9.98	8.15	11.0	21.9	20.5	23.0	18.0	14.7	20.6
Mean cumulative sleep score ± SD	1.03 ± 1.15	0.966 ± 1.12	1.07 ± 1.17	0.958 ± 1.12	0.883 ± 1.07	1.00 ± 1.14	1.54 ± 1.27	1.49 ± 1.25	1.59 ± 1.28	1.38 ± 1.23	1.24 ± 1.20	1.50 ± 1.25
Sleep medication use (yes)	23.7	19.9	25.9	24.5	20.4	26.7	16.3	15.2	17.2	22.1	20.0	23.8
*Clinical characteristics*												
Mean BMI ± SD (kg/m^2^)	27.6 ± 6.12	27.3 ± 6.30	27.8 ± 6.00	27.2 ± 5.94	26.8 ± 6.00	27.5 ± 5.88	31.2 ± 6.93	31.1 ± 7.46	31.2 ± 6.50	28.2 ± 5.73	27.9 ± 5.92	28.4 ± 5.57
Underweight (< 18.5 kg/m^2^)	1.12	1.19	1.09	1.22	1.31	1.18	0.37	0.49	0.27	0.65	0.61	0.68
Normal weight (18.5–24.9 kg/m^2^)	38.3	42.7	35.7	40.8	45.9	38.0	17.0	19.8	14.7	31.4	36.5	27.3
Overweight (25.0–29.9 kg/m^2^)	31.6	29.0	33.1	31.2	28.4	32.8	31.8	30.8	32.6	37.4	33.3	31.4
Obese (≥ 30.0 kg/m^2^)	29.0	27.1	30.1	26.8	24.3	28.1	50.9	48.8	52.4	30.6	29.6	31.4
Physician-diagnosed clinical depression or bipolar disorder (yes)	19.9	18.9	20.5	20.2	19.5	20.6	15.3	13.5	16.7	22.5	18.9	25.3
Hormone replacement therapy use (yes)	10.3	11.7	14.4	10.9	3.44	15.0	6.85	2.33	10.4	6.09	2.33	9.07
Postmenopausal (yes)	63.2	–	100	64.3	0	100	56.3	0	100	55.7	0	100
Natural menopause (yes)	63.9	–	63.9	65.2	–	65.2	50.3	–	50.3	61.2	–	61.2
Use of lipid control medications (yes)	18.4	7.45	24.8	18.5	7.23	24.7	17.9	8.54	25.2	18.5	8.72	26.2
Metabolic Abnormalities, median (IQR)	1 (0–2)	1 (0–1)	1 (0–2)	1 (0–2)	0 (0–1)	1 (0–2)	2 (1–2)	1 (0–2)	2 (1–3)	1 (0–2)	1 (0–1)	1 (1–2)
Hypertension as defined by original definition (yes)^B^	30.3	18.5	37.2	28.5	16.0	35.4	49.5	39.2	57.5	29.6	18.7	38.3
Hypertension as defined by new guidelines (yes)^B^	38.4	27.0	45.1	36.6	24.4	43.3	57.8	48.4	65.0	37.8	26.9	46.4
Abdominal obesity (yes)	39.4	35.0	42.0	37.2	32.4	39.9	59.1	54.3	62.9	43.8	38.9	47.6
Dyslipidemia (yes)	32.8	19.3	40.7	32.4	18.4	40.1	33.8	22.9	42.2	38.7	24.7	59.9
Prediabetes or type 2 diabetes mellitus (yes)	6.99	4.28	8.56	6.00	3.47	7.40	14.3	8.82	18.5	12.0	8.11	15.0
Metabolic Abnormalities consistent with MetS (yes)^C^	11.9	5.63	15.5	10.8	4.60	14.2	21.3	12.4	28.2	15.4	8.72	20.8
Metabolic Abnormalities consistent with MetS (yes)^D^	13.3	6.68	17.1	12.1	35.7	64.3	22.6	13.3	29.9	17.0	9.58	22.9

Data presented as %, median and IQR, or mean ± SD. Percentages may not sum to 100 due to rounding

Proportion employed is calculated as: number employed/(number employed + unemployed + homemaker + student + retired). Healthy Eating Index scores range from 0 to 100 with higher scores indicating a healthier diet [38]. Proportion of natural menopause is calculated as: number reporting natural menopause/all women reporting menopause

Inconsistent weekly sleep patterns indicated whether participants reported consistent (could vary day-by-day but were stable from week-to-week) or inconsistent wake-up times and bedtimes during the prior 6 weeks. Sleep debt was defined as ≥ 2-h difference between average longest and shortest sleep duration. Insomnia symptoms included difficulty falling asleep, defined as taking > 30 min vs. ≤ 30 min to fall asleep on average, or difficulty staying asleep, defined as waking up ≥ 3 times per night ≥ 3 nights/week vs. < 3 times per night < 3 nights/week vs. neither. Cumulative sleep score was the sum of yes responses to the main sleep characteristics (short sleep duration, inconsistent weekly sleep patterns, sleep debt, frequent napping, and insomnia symptoms [range: 0–5])

Missingness: < 0.2% for household size, number of children in household, marital status, BMI/BMI category, glycemic load, sleep medication use, habitual sleep duration category, consistent sleep pattern, sleep debt, napping, insomnia symptoms, difficulty falling asleep, difficulty staying asleep, cumulative sleep score; 0% for age, educational attainment, current employment, annual household income, smoking status, alcohol consumption, METs-hours/week, Healthy Eating Index Score, clinical depression/bipolar disorder, hormone replacement therapy use, menopausal status, number of baseline metabolic abnormalities/MetS, hypertension, abdominal obesity, dyslipidemia, borderline/type 2 diabetes mellitus

*SD* standard deviation, *IQR* interquartile range, *BMI* body mass index, *METs* metabolic equivalent, *MetS* metabolic syndrome

^A^Participants who reported long sleep duration (> 9 h) were excluded due to small sample size (n = 349)

^B^Original definition (systolic blood pressure (blood pressure) > 130 mmHg or diastolic BP > 85 mmHg) as defined by Alberti et al. [35] and new guidelines refer to the statement of the American College of Cardiology/American Heart Association Task Force (2017) (systolic BP > 130 mmHg or diastolic BP > 80 mmHg)

^C^Metabolic abnormalities consistent with MetS were based on an adapted version of the harmonized definition for MetS [35]: the presence of three or more individual metabolic abnormalities (i.e., hypertension, abdominal obesity, dyslipidemia, hyperglycemia)

^D^Adapted harmonized definition of MetS applying the American College of Cardiology/American Heart Association Task Force (2017) guidelines for hypertension [45]

### Poor sleep and MetS by menopausal status and race/ethnicity

**Fig. 1:**
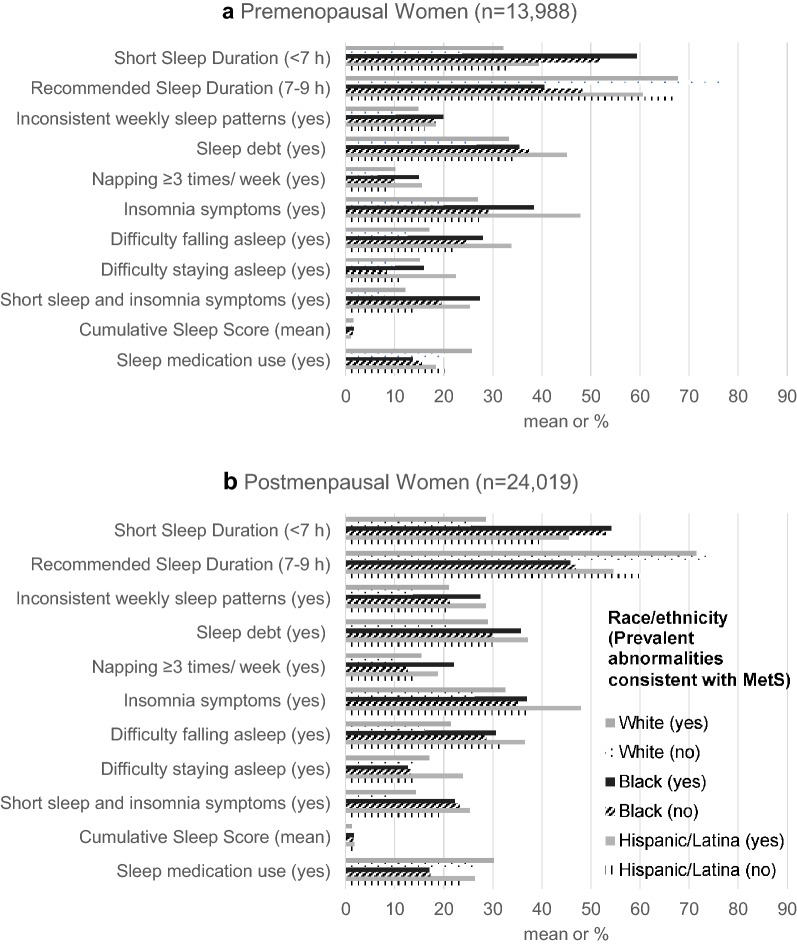
Prevalence of sleep characteristics by race/ethnicity and metabolic abnormalities consistent with metabolic syndrome (MetS) among **a** premenopausal and **b** postmenopausal participants, Sister Study (2003–2009), N = 38,007. *h* hours. Data presented as percentages of participants reporting each sleep characteristics and mean (of cumulative sleep score) within each race/ethnicity/MetS category. Inconsistent weekly sleep patterns indicated whether participants reported consistent (could vary day-by-day but were stable from week-to-week) or inconsistent wake-up times and bedtimes during the prior 6 weeks. Sleep debt was defined as ≥ 2-h difference between average longest and shortest sleep duration. Insomnia symptoms included difficulty falling asleep, defined as taking > 30 min vs. ≤ 30 min to fall asleep on average, or difficulty staying asleep, defined as waking up ≥ 3 times per night ≥ 3 nights/week vs. < 3 times per night < 3 nights/week vs. neither. Cumulative sleep score was the sum of yes responses to the main sleep characteristics (short sleep duration, inconsistent weekly sleep patterns, sleep debt, frequent napping, and insomnia symptoms [range: 0–5]). < 0.2% missingness for sleep medication use, habitual sleep duration category, consistent sleep pattern, sleep debt, napping, insomnia symptoms, difficulty falling asleep, difficulty staying asleep, cumulative sleep score

Women with prevalent MetS generally had a higher prevalence of poor sleep characteristics regardless of race/ethnicity compared to women without MetS; however, the differences in MetS prevalence among postmenopausal women were often smaller than those observed among premenopausal women, particularly among blacks (Fig. 1). After adjusting for potential confounders, associations between certain poor sleep characteristics, including *short sleep duration, insomnia symptoms*, *difficulty staying asleep*, concurrent *short sleep duration and insomnia symptoms*, and MetS were stronger among premenopausal compared to postmenopausal women; however, positive associations were similar regardless of menopausal status for *inconsistent weekly sleep patterns*, *sleep debt, napping*, *difficulty falling asleep,* and *sleep score* (Table 2). Premenopausal women reporting *short sleep* duration had 23% higher prevalence of MetS (PR = 1.23 [95% CI 1.06–1.42]) compared to their counterparts reporting recommended sleep duration, but there was 9% higher prevalence of MetS among postmenopausal women who reported short sleep (PR = 1.09 [1.02–1.16], p_short sleep*menopause_ = 0.0070). Compared to counterparts without *insomnia symptoms*, premenopausal women with *insomnia symptoms* had a 21% higher prevalence of MetS (PR = 1.21 [1.05–1.41]) and postmenopausal women with *insomnia symptoms* had a 11% higher prevalence of MetS (PR = 1.11 [1.05–1.18], p_insomnia symptoms*menopause_ = 0.035). Although relationships between *difficulty falling asleep* and MetS did not vary by menopausal status, *difficulty staying asleep* varied by menopausal status: *difficulty staying asleep* was associated with a 33% higher prevalence of MetS among premenopausal women (PR = 1.33 [1.11–1.59]) but was not associated with MetS among postmenopausal women (PR = 1.07 [0.99–1.16], p_difficulty staying asleep*menopause_ = 0.0074). Only relationships for *short sleep duration* and *insomnia symptoms* varied by menopausal status (PR_premenopausal_ = 1.25 [1.04–1.50], PR_postmenopausal_ = 1.12 [1.03–1.21], p_short sleep duration/insomnia symptoms*menopause_ = 0.027) and race/ethnicity (p_short sleep duration/insomnia symptoms*race/ethnicity_ < 0.05). White and Hispanic/Latina women who reported concurrent *short sleep duration* and *insomnia symptoms* had higher prevalence of MetS compared to their within-race counterparts; however, there was no association among black menopausal women.

**Table 2:** Adjusted prevalence ratios for metabolic abnormalities consistent with metabolic syndrome for pre- and post-menopausal women with poor sleep compared to women with recommended sleep, sister study (2003–2009), N = 38,007

	Total	White	Black	Hispanic
*Premenopausal women*				
Sample size	N = 13,988	n = 11,757	n = 1417	n = 814
n (%) with prevalent abnormalities consistent with MetS	787 (5.63)	541 (4.60)	175 (12.4)	71 (8.72)
	PR (95% CI)			
Short sleep duration (< 7 h vs. recommended [7–9 h])	*1.23 (1.06–1.42)*	*1.26 (1.06–1.50)*	1.18 (0.89–1.57)	NE
Inconsistent weekly sleep patterns (yes vs. no)	1.05 (0.87–1.26)	1.14 (0.90–1.43)	0.98 (0.68–1.40)	NE
Sleep debt (yes vs. no)	1.16 (0.99–1.35)	1.16 (0.95–1.42)	0.91 (0.67–1.24)	NE
Napping ≥ 3 times/week (yes vs. no)	*1.25 (1.01–1.56)*	1.18 (0.90–1.56)	1.35 (0.91–1.98)	NE
Insomnia symptoms (yes vs. no)	*1.21 (1.05–1.41)*	1.10 (0.91–1.33)	1.32 (0.99–1.75)	NE
Difficulty falling asleep (yes vs. no)	1.11 (0.93–1.32)	1.05 (0.83–1.32)	1.13 (0.82–1.56)	NE
Difficulty staying asleep (yes vs. no)	*1.33 (1.11–1.59)*	1.21 (0.97–1.51)	*1.52 (1.06–2.18)*	NE
Short sleep and insomnia symptoms (yes vs. no)	*1.25 (1.04–1.50)*	1.17 (0.91–1.50)	1.28 (0.94–1.75)	NE
Cumulative sleep score	*1.10 (1.04–1.16)*	*1.10 (1.03–1.17)*	1.08 (0.96–1.20)	NE
*Postmenopausal women*				
Sample size	N = 24,019	n = 21,168	n = 1826	n = 1025
n (%) with prevalent abnormalities consistent with MetS	3725 (15.5)	2998 (14.2)	514 (28.2)	213 (20.8)
	PR (95% CI)			
Short sleep duration (< 7 h vs. recommended [7–9 h])	*1.09 (1.02–1.16)*	*1.09 (1.01–1.17)*	1.05 (0.91–1.22)	1.23 (0.97–1.55)
Inconsistent weekly sleep patterns (yes vs. no)^B^	*1.23 (1.15–1.31)*	*1.26 (1.17–1.36)*	1.11 (0.94–1.30)	1.17 (0.89–1.53)
Sleep debt (yes vs. no)	*1.24 (1.14–1.35)*	*1.25 (1.13–1.38)*	1.15 (0.96–1.39)	1.21 (0.91–1.62)
Napping ≥ 3 times/week (yes vs. no)	*1.27 (1.18–1.37)*	*1.27 (1.16–1.39)*	*1.34 (1.13–1.58)*	1.17 (0.88–1.55)
Insomnia symptoms (yes vs. no)	*1.11 (1.05–1.18)*	*1.12 (1.05–1.21)*	1.00 (0.86–1.16)	1.25 (0.97–1.61)
Difficulty falling asleep (yes vs. no)	*1.11 (1.04–1.19)*	*1.14 (1.05–1.23)*	1.02 (0.87–1.20)	1.10 (0.84–1.42)
Difficulty staying asleep (yes vs. no)^B^	1.07 (0.99–1.16)	1.08 (0.99–1.17)	0.91 (0.73–1.13)	*1.38 (1.06–1.81)*
Short sleep and insomnia symptoms (yes vs. no)^A^	*1.12 (1.03–1.21)*	*1.17 (1.07–1.28)*	0.92 (0.77–1.10)	1.20 (0.91–1.59)
Cumulative sleep score^B^	*1.11 (1.08–1.13)*	*1.11 (1.08–1.14)*	*1.07 (1.01–1.13)*	*1.14 (1.04–1.25)*

*P*_short sleep*menopausal status_ = 0.0070; *P*_consistent sleep*menopausal status_ = 0.71; *P*_sleep debt*menopausal status_ = 0.27; *P*_napping*menopausal status_ = 0.38; *P*_insomnia symptoms*menopausal status_ = 0.035; *P*_difficulty falling asleep*menopausal status_ = 0.32; *P*_difficulty staying asleep*menopausal status_ = 0.0074; *P*_short sleep and insomnia symptoms*menopausal status_ = 0.027; *P*_sleep score *menopausal status_ = 0.15

Adjusted for age at baseline (years), educational attainment (≤ high school graduate/graduation equivalent degree, some college/technical school/associate’s degree, ≥ college graduate), annual household income (< $20,000, $20,000–$49,999, $50,000–$99,999, ≥ $100,000), diet quality (Healthy Eating Index score [38]), physical activity (METs [metabolic equivalent] hours per week), use of hormone replacement therapy (yes vs. no), alcohol consumption (nondrinker [former/never], light/moderate [≤ 7 drinks/week], heavy [> 7 drinks/week]), smoking status (never, former, current), clinical depression or bipolar disorder (yes vs. no), and sleep medication use (yes vs. no). Models for sleep debt are also adjusted for consistent weekly sleep patterns (no vs. yes)

Inconsistent weekly sleep patterns indicated whether participants reported consistent (could vary day-by-day but were stable from week-to-week) or inconsistent wake-up times and bedtimes during the prior 6 weeks. Sleep debt was defined as ≥ 2-h difference between average longest and shortest sleep duration. Insomnia symptoms included difficulty falling asleep, defined as taking > 30 min vs. ≤ 30 min to fall asleep on average, or difficulty staying asleep, defined as waking up ≥ 3 times per night ≥ 3 nights/week vs. < 3 times per night < 3 nights/week vs. neither. Cumulative sleep score was the sum of yes responses to the main sleep characteristics (short sleep duration, inconsistent weekly sleep patterns, sleep debt, frequent napping, and insomnia symptoms [range: 0–5])

< 0.2% missingness for sleep medication use, habitual sleep duration category, consistent sleep pattern, sleep debt, napping, insomnia symptoms, difficulty falling asleep, difficulty staying asleep, cumulative sleep score

*MetS* metabolic syndrome, *PR* prevalence ratio, *CI* confidence interval, *h* hours, *NE* not estimable

Italic values indicate statistical significance at two-sided p = 0.05

^A^p < 0.05 for interaction term (sleep variable by race/ethnicity)

^B^p < 0.10 for interaction term (sleep variable by race/ethnicity)

Results of the sensitivity analyses are presented in Additional file 1: Tables S1–S9. Although prevalence ratios were slightly attenuated, overall findings were consistent after adjustment for use of lipid-regulating medications (Additional file 1: Table S1). Among all postmenopausal women, associations between sleep characteristics and MetS were similar between women who reported natural and non-natural menopause (Additional file 1: Table S2). After application of an 80 mmHg cut-point for hypertension, associations between sleep characteristics and MetS prevalence were consistent with results for original BP cut-points (Additional file 1: Table S3). Among premenopausal women, PR’s for most poor sleep characteristics were higher when the analysis was restricted to participants with no sleep medication use; however, among all postmenopausal women, PR’s were comparable irrespective of sleep medication use (Additional file 1: Table S4). Nonetheless, data suggested some variation by sleep medication use among blacks and Hispanics/Latinas. After mutual adjustment for individual sleep characteristics in models, results for short sleep duration were robust for *short sleep duration* and *insomnia symptoms:* each was more strongly associated with MetS among premenopausal women (Additional file 1: Table S5). Consistently, associations between *short sleep duration*, *insomnia*-*related symptoms,* and most individual MetS components (hypertension, abdominal obesity, and dyslipidemia) were stronger among premenopausal compared to postmenopausal women (Additional file 1: Tables S6–S9). Notably, associations between poor sleep and abdominal obesity were often strongest for whites among premenopausal women and strongest for Hispanics/Latinas among postmenopausal women (Additional file 1: Table S7).

## Discussion

In this large study of white, black, and Hispanic/Latina women aged 35–74 years, short sleep duration and insomnia symptoms were positively associated with prevalent MetS. These associations were stronger among premenopausal compared to postmenopausal women. Furthermore, among postmenopausal women, relationships between concurrent short sleep and insomnia symptoms and MetS were stronger among Hispanics/Latinas and whites. We observed no other racial/ethnic differences in the relationship between sleep and MetS. Pre- and post-menopausal women who reported no consistent weekly sleep pattern, sleep debt, frequent napping, and difficulty falling asleep had similarly higher prevalence of MetS. Our findings were robust after sensitivity analyses.

Our finding that poor sleep characteristics were associated with greater MetS prevalence is consistent with prior studies. Meta-analyses and a prospective study reported positive associations between short sleep duration and MetS as well as a dose–response relationship where odds of MetS increased with decreases in sleep duration [6, 7, 17, 31, 32]. Similarly, several cross-sectional studies across geographically and ethnically diverse populations observed positive relationships between poor sleep, including sleep disturbances and difficulty staying asleep, and prevalent MetS [9–11, 13]. These prior studies often adjusted for menopausal status, but none explicitly investigated menopausal status as a potential modifier or presented results for premenopausal and postmenopausal participants, separately. Counter to our observations, cross-sectional studies of participants in Spain, Japan, and China as well as a prospective study among black and white men and women aged 45–74 years in Pittsburgh observed relationships between difficulty falling asleep and MetS [11–14]. The conflicting results could be due to differences in population characteristics and measurement of sleep and MetS. However, our results remain biologically plausible.

Poor sleep is hypothesized to affect each individual component of MetS through several distinct biological pathways [18, 24]. For instance, lack of adequate sleep can alter homeostatic functioning, result in greater caloric intake by increasing appetite through dysregulation of hormones that increase hunger and decrease satiety, and lead to lower physical activity due to fatigue [18, 24]. Poor sleep can also affect glucose homeostasis and experimental evidence has shown that sleep restriction can induce insulin resistance likely through inflammatory pathways and epigenetic changes to the expression of circadian clock genes that regulate biological processes [18, 24]. Furthermore, sleep restriction raises blood pressure through multiple potential mechanisms like higher catecholamine production and hyperactivity of the sympathetic nervous system [18]. Subjective sleep characteristics are likely reflective of inadequate sleep and sleep deprivation, and these pathways could explain our observations. Nonetheless, objective measures of sleep and MetS coupled with biomarkers of physiological functioning are necessary.

Our finding that poor sleep was more strongly associated with MetS among premenopausal compared to postmenopausal women may be due to biological differences between premenopausal and postmenopausal women. The menopausal transition negatively affects sleep and leads to increased insomnia, which tends to continue post-transition [28]. Additionally, postmenopausal women have greater cardiovascular disease risk compared to premenopausal women likely due to the decline of sex hormones, namely estrogen [29, 30]. Prior studies have also shown higher prevalence of sleep-disordered breathing (SDB) among postmenopausal women compared to premenopausal women [33, 46]. Prevalent SDB among postmenopausal women could explain higher prevalence of both poor sleep and MetS observed in this group in our cross-sectional study. It is possible that the greater likelihood of both poor sleep and MetS among postmenopausal women results in weaker relative associations compared to premenopausal women.

Among postmenopausal women, our findings that associations between concurrent short sleep duration/insomnia symptoms and MetS were stronger among Hispanic/Latina and white postmenopausal women compared to their black counterparts, require additional investigation. Data suggested that associations between concurrent short sleep/insomnia symptoms and the MetS component abdominal obesity were strongest among Hispanics/Latinas. Our MetS findings may have captured racial/ethnic differences in associations between sleep and abdominal obesity. Recent prior studies consisting of similarly aged US women lacked Hispanic/Latina participants [8, 14]. More racially/ethnically diverse prospective studies are warranted.

This study has limitations as well as important strengths. This study was limited by its cross-sectional study design, which precluded our ability to infer causal relationships between poor sleep and MetS. Secondly, we relied on self-reported measures for sleep characteristics, dyslipidemia, and borderline/T2DM; however, all women were of higher SES and racial/ethnic differences in health care access and utilization necessary for diagnoses would be it is less likely. Self-reported sleep duration has been shown to be overestimated compared to objectively-measured sleep duration through wrist actigraphy and polysomnography [47]. Although measurement error could vary by race/ethnicity, it is generally non-differential and likely leads to underestimation of relationships between poor sleep and MetS among all participants [48]. Furthermore, sleep characteristics were not assessed using standardized questionnaires; as a result, our assessment of sleep characteristics may be limited due to lack of psychometric validation. We also lacked data on SDB and sleep apnea, a sleep disorder that could affect measured sleep characteristics and MetS as well as partially explain observed associations, particularly between difficulty staying asleep and MetS [8, 14]. Therefore, future studies inclusive of clinical diagnoses of MetS and objective sleep measures including sleep apnea are necessary. Thirdly, we did not study perimenopause; but, future studies should consider perimenopause [29]. Fourth, the lower sample size among premenopausal women and non-whites resulted in limited power to estimate relationships and to test effect modification. Future research with greater diversity and consideration of ethnic subgroups is needed as sleep characteristics have been shown to differ by ethnic group [26]. Lastly, due to the testing of multiple associations, results could be due to chance. Despite these limitations, we used data from a large cohort of US women, which allowed for stratification by menopausal status and race/ethnicity. We also investigated multiple sleep characteristics beyond sleep duration, included objective measures for abdominal obesity and hypertension, and adjusted for many potential confounders. Furthermore, our results were robust as demonstrated by the consistent relationships observed in sensitivity analysis.

## Conclusions

In this study of a multi-ethnic population of middle-aged white, black, and Hispanic/Latina women, we found that multiple poor sleep characteristics were associated with higher prevalence of MetS. Beyond a weaker association between combined short sleep duration/insomnia symptoms and MetS among black women, relationships between poor sleep and MetS did not vary by race/ethnicity. Nonetheless, relationships between short sleep duration as well as insomnia symptoms and MetS were stronger among premenopausal compared to postmenopausal women. Our results have public health and clinical significance, especially in the context of primary and secondary prevention of MetS. Promotion of good sleep hygiene like acquiring the recommended amount of sleep and addressing insomnia symptoms throughout the lifecourse may be a worthwhile approach to the prevention of poor cardiometabolic health among women, especially prior to the menopausal transition. Nonetheless, future research with sufficient representation of racial/ethnic minorities, longitudinal study designs, and objective measures are warranted to corroborate our findings.

## Additional file

**Additional file 1: Figure S1.** Composition of Analytic Sample. **Table S1.** Additionally Adjusted (for Use of Medication for Dyslipidemia) Prevalence Ratios for Metabolic Abnormalities Consistent with Metabolic Syndrome for Pre- and Post-Menopausal Women with Poor Sleep Compared to Women with Recommended Sleep, Sister Study (2003-2009), N = 38,007. **Table S2.** Adjusted Prevalence Ratios of Metabolic Abnormalities Consistent with Metabolic Syndrome for Poor Sleep Compared to Recommended Sleep among Postmenopausal Women, Stratified by Type of Menopause, Sister Study (2003-2009), N = 24,019. **Table S3.** Adjusted Prevalence Ratios of Metabolic Abnormalities Consistent with Metabolic Syndrome for Pre- and Post-Menopausal Women with Poor Sleep Compared to Women with Recommended Sleep using Elevated Diastolic Blood Pressure Cut Point of ≥ 80 mmHg, Sister Study (2003-2009), N = 38,007. **Table S4.** Adjusted Prevalence Ratios of Metabolic Abnormalities Consistent with Metabolic Syndrome for Pre- and Post-Menopausal Women with Poor Sleep Compared to Women with Recommended Sleep Stratified by Sleep Medication Use, Sister Study (2003-2009), N = 38,007. **Table S5.** Additionally Adjusted (for other sleep characteristics) Prevalence Ratios of Metabolic Abnormalities Consistent with Metabolic Syndrome for Pre- and Post-Menopausal Women with Poor Sleep Compared to Women with Recommended Sleep, Sister Study (2003-2009), N = 38,007. **Table S6.** Adjusted Prevalence Ratios of Hypertension for Pre- and Post-Menopausal Women with Poor Sleep Compared to Women with Recommended Sleep, Sister Study (2003-2009), N = 38,007. **Table S7.** Adjusted Prevalence Ratios of Abdominal Obesity for Pre- and Post-Menopausal Women with Poor Sleep Compared to Women with Recommended Sleep, Sister Study (2003-2009), N = 38,007. **Table S8.** Adjusted Prevalence Ratios of Dyslipidemia for Pre- and Post-Menopausal Women with Poor Sleep Compared to Women with Recommended Sleep, Sister Study (2003-2009), N = 38,007. **Table S9.** Adjusted Prevalence Ratios of Prediabetes/Type 2 Diabetes Mellitus (T2DM) for Pre- and Post-Menopausal Women with Poor Sleep Compared to Women with Recommended Sleep, Sister Study (2003-2009), N=38,007.

## Abbreviations

MetS: metabolic syndrome
BP: blood pressure
T2DM: type 2 diabetes mellitus
HEI: healthy eating index
SDB: sleep-disordered breathing

## References

[1] Reaven GM (1993). Role of insulin resistance in human disease (syndrome X): an expanded definition. Annu Rev Med.

[2] Mottillo S, Filion KB, Genest J, Joseph L, Pilote L, Poirier P (2010). The metabolic syndrome and cardiovascular risk a systematic review and meta-analysis. J Am Coll Cardiol.

[3] Moore JX, Chaudhary N, Akinyemiju T (2017). Metabolic syndrome prevalence by race/ethnicity and sex in the united states, national health and nutrition examination survey, 1988–2012. Prev Chronic Dis.

[4] Benjamin EJ, Blaha MJ, Chiuve SE, Cushman M, Das SR, Deo R (2017). Heart disease and stroke statistics-2017 update: a report from the American heart association. Circulation.

[5] Cappuccio FP, Taggart FM, Kandala NB, Currie A, Peile E, Stranges S (2008). Meta-analysis of short sleep duration and obesity in children and adults. Sleep.

[6] Iftikhar IH, Donley MA, Mindel J, Pleister A, Soriano S, Magalang UJ (2015). Sleep duration and metabolic syndrome. An updated dose-risk metaanalysis. Ann Am Thorac Soc.

[7] Xi B, He D, Zhang M, Xue J, Zhou D (2014). Short sleep duration predicts risk of metabolic syndrome: a systematic review and meta-analysis. Sleep Med Rev.

[8] Hall MH, Okun ML, Sowers M, Matthews KA, Kravitz HM, Hardin K (2012). Sleep is associated with the metabolic syndrome in a multi-ethnic cohort of midlife women: the SWAN sleep study. Sleep.

[9] Hung HC, Yang YC, Ou HY, Wu JS, Lu FH, Chang CJ (2013). The association between self-reported sleep quality and metabolic syndrome. PLoS ONE.

[10] Lee J, Choi YS, Jeong YJ, Lee J, Kim JH, Kim SH (2013). Poor-quality sleep is associated with metabolic syndrome in Korean adults. Tohoku J Exp Med.

[11] Lin SC, Sun CA, You SL, Hwang LC, Liang CY, Yang T (2016). The link of self-reported insomnia symptoms and sleep duration with metabolic syndrome: a chinese population-based study. Sleep.

[12] Mesas AE, Guallar-Castillon P, Lopez-Garcia E, Leon-Munoz LM, Graciani A, Banegas JR (2014). Sleep quality and the metabolic syndrome: the role of sleep duration and lifestyle. Diabetes Metab Res Rev.

[13] Okubo N, Matsuzaka M, Takahashi I, Sawada K, Sato S, Akimoto N (2014). Relationship between self-reported sleep quality and metabolic syndrome in general population. BMC Public Health.

[14] Troxel WM, Buysse DJ, Matthews KA, Kip KE, Strollo PJ, Hall M (2010). Sleep symptoms predict the development of the metabolic syndrome. Sleep.

[15] Yoo H, Franke WD (2013). Sleep habits, mental health, and the metabolic syndrome in law enforcement officers. J Occup Environ Med.

[16] Zohal M, Ghorbani A, Esmailzadehha N, Ziaee A, Mohammadi Z (2017). Association of sleep quality components and wake time with metabolic syndrome: the qazvin metabolic diseases study (QMDS), Iran. Diabetes Metab Syndr.

[17] Kim JY, Yadav D, Ahn SV, Koh SB, Park JT, Yoon J (2015). A prospective study of total sleep duration and incident metabolic syndrome: the ARIRANG study. Sleep Med.

[18] Koren D, Dumin M, Gozal D (2016). Role of sleep quality in the metabolic syndrome. Diabetes Metab Syndr Obes.

[19] Buxton OM, Cain SW, O’Connor SP, Porter JH, Duffy JF, Wang W (2012). Adverse metabolic consequences in humans of prolonged sleep restriction combined with circadian disruption. Sci Transl Med.

[20] Van Cauter E, Spiegel K, Tasali E, Leproult R (2008). Metabolic consequences of sleep and sleep loss. Sleep Med.

[21] Liu Y, Wheaton AG, Chapman DP, Cunningham TJ, Lu H, Croft JB (2016). Prevalence of healthy sleep duration among adults-United States, 2014. MMWR Morb Mortal Wkly Rep.

[22] Hirshkowitz M, Whiton K, Albert SM, Alessi C, Bruni O, DonCarlos L (2015). National sleep foundation’s updated sleep duration recommendations: final report. Sleep Health.

[23] NCHS (2017). Health, United States, Health, United State6: with chartbook on long-term trends in health.

[24] Jackson CL, Redline S, Emmons KM (2015). Sleep as a potential fundamental contributor to disparities in cardiovascular health. Annu Rev Public Health.

[25] Jackson CL (2017). Determinants of racial/ethnic disparities in disordered sleep and obesity. Sleep Health.

[26] Grandner MA, Williams NJ, Knutson KL, Roberts D, Jean-Louis G (2016). Sleep disparity, race/ethnicity, and socioeconomic position. Sleep Med.

[27] Freedman RR (2014). Menopause and sleep. Menopause.

[28] Baker FC, de Zambotti M, Colrain IM, Bei B (2018). Sleep problems during the menopausal transition: prevalence, impact, and management challenges. Nat Sci Sleep.

[29] Pu D, Tan R, Yu Q, Wu J (2017). Metabolic syndrome in menopause and associated factors: a meta-analysis. Climacteric.

[30] van Dijk GM, Kavousi M, Troup J, Franco OH (2015). Health issues for menopausal women: the top 11 conditions have common solutions. Maturitas.

[31] Chaput JP, McNeil J, Despres JP, Bouchard C, Tremblay A (2013). Seven to eight hours of sleep a night is associated with a lower prevalence of the metabolic syndrome and reduced overall cardiometabolic risk in adults. PLoS ONE.

[32] Choi JK, Kim MY, Kim JK, Park JK, Oh SS, Koh SB (2011). Association between short sleep duration and high incidence of metabolic syndrome in midlife women. Tohoku J Exp Med.

[33] Heinzer R, Marti-Soler H, Marques-Vidal P, Tobback N, Andries D, Waeber G (2018). Impact of sex and menopausal status on the prevalence, clinical presentation, and comorbidities of sleep-disordered breathing. Sleep Med.

[34] Sandler DP, Hodgson ME, Deming-Halverson SL, Juras PS, D’Aloisio AA, Suarez LM (2017). The sister study cohort: baseline methods and participant characteristics. Environ Health Perspect.

[35] Alberti KG, Eckel RH, Grundy SM, Zimmet PZ, Cleeman JI, Donato KA (2009). Harmonizing the metabolic syndrome: a joint interim statement of the international diabetes federation task force on epidemiology and prevention; national heart, lung, and blood institute; American heart association; world heart federation; international atherosclerosis society; and international association for the study of obesity. Circulation.

[36] Ainsworth BE, Haskell WL, Whitt MC, Irwin ML, Swartz AM, Strath SJ (2000). Compendium of physical activities: an update of activity codes and MET intensities. Med Sci Sports Exerc.

[37] Ainsworth BE, Haskell WL, Leon AS, Jacobs DR, Montoye HJ, Sallis JF (1993). Compendium of physical activities: classification of energy costs of human physical activities. Med Sci Sports Exerc.

[38] Guenther PM, Kirkpatrick SI, Reedy J, Krebs-Smith SM, Buckman DW, Dodd KW (2014). The healthy eating index-2010 is a valid and reliable measure of diet quality according to the 2010 guidelines dietary for Americans. J Nutr.

[39] Atkinson FS, Foster-Powell K, Brand-Miller JC (2008). International tables of glycemic index and glycemic load values: 2008. Diabetes Care.

[40] Block G, Hartman AM, Dresser CM, Carroll MD, Gannon J, Gardner L (1986). A data-based approach to diet questionnaire design and testing. Am J Epidemiol.

[41] USPSTF (2004). Screening for obesity in adults: recommendations and rationale. Am Fam Physician.

[42] Grandner MA, Patel NP, Gehrman PR, Xie D, Sha D, Weaver T (2010). Who gets the best sleep? Ethnic and socioeconomic factors related to sleep complaints. Sleep Med.

[43] Barros AJ, Hirakata VN (2003). Alternatives for logistic regression in cross-sectional studies: an empirical comparison of models that directly estimate the prevalence ratio. BMC Med Res Methodol.

[44] Greenland S, Pearl J, Robins JM (1999). Casual diagrams for epidemiologic research. Epidemiology.

[45] Whelton PK, Carey RM, Aronow WS, Casey DE, Collins KJ, Dennison Himmelfarb C (2018). 2017 ACC/AHA/AAPA/ABC/ACPM/AGS/APhA/ASH/ASPC/NMA/PCNA guideline for the prevention, detection, evaluation, and management of high blood pressure in adults. A Report of the American college of cardiology/American heart association task force on clinical practice guidelines. Hypertension.

[46] Gomez-Santos C, Saura CB, Lucas JA, Castell P, Madrid JA, Garaulet M (2016). Menopause status is associated with circadian- and sleep-related alterations. Menopause.

[47] Lauderdale DS, Knutson KL, Yan LL, Liu K, Rathouz PJ (2008). Self-reported and measured sleep duration: how similar are they?. Epidemiology.

[48] Jackson CL, Patel SR, Jackson WB, Lutsey PL, Redline S (2018). Agreement between self-reported and objectively measured sleep duration among white, black, Hispanic, and Chinese adults in the United States: multi-ethnic study of atherosclerosis. Sleep.

