# Supplementary material for: Multiple poor sleep characteristics and metabolic abnormalities consistent with metabolic syndrome among white, black, and Hispanic/Latina women: modification by menopausal status
Source: Diabetol Metab Syndr. 2019 Feb 14;11:17. doi: 10.1186/s13098-019-0413-2 (PMC6376679; doi:10.1186/s13098-019-0413-2)
Supplement: Supplementary file 1 — Additional file 1: Figure S1. Composition of Analytic Sample. Table S1. Additionally Adjusted (for Use of Medication for Dyslipidemia) Prevalence Ratios for Metabolic Abnormalities Consistent with Metabolic Syndrome for Pre- and Post-Menopausal Women with Poor Sleep Compared to Women with Recommended Sleep, Sister Study (2003-2009), N = 38,007. Table S2. Adjusted Prevalence Ratios of Metabolic Abnormalities Consistent with Metabolic Syndrome for Poor Sleep Compared to Recommended Sleep among Postmenopausal Women, Stratified by Type of Menopause, Sister Study (2003-2009), N = 24,019. Table S3. Adjusted Prevalence Ratios of Metabolic Abnormalities Consistent with Metabolic Syndrome for Pre- and Post-Menopausal Women with Poor Sleep Compared to Women with Recommended Sleep using Elevated Diastolic Blood Pressure Cut Point of ≥ 80 mmHg, Sister Study (2003-2009), N = 38,007. Table S4. Adjusted Prevalence Ratios of Metabolic Abnormalities Consistent with Metabolic Syndrome for Pre- and Post-Menopausal Women with Poor Sleep Compared to Women with Recommended Sleep Stratified by Sleep Medication Use, Sister Study (2003-2009), N = 38,007. Table S5. Additionally Adjusted (for other sleep characteristics) Prevalence Ratios of Metabolic Abnormalities Consistent with Metabolic Syndrome for Pre- and Post-Menopausal Women with Poor Sleep Compared to Women with Recommended Sleep, Sister Study (2003-2009), N = 38,007. Table S6. Adjusted Prevalence Ratios of Hypertension for Pre- and Post-Menopausal Women with Poor Sleep Compared to Women with Recommended Sleep, Sister Study (2003-2009), N = 38,007. Table S7. Adjusted Prevalence Ratios of Abdominal Obesity for Pre- and Post-Menopausal Women with Poor Sleep Compared to Women with Recommended Sleep, Sister Study (2003-2009), N = 38,007. Table S8. Adjusted Prevalence Ratios of Dyslipidemia for Pre- and Post-Menopausal Women with Poor Sleep Compared to Women with Recommended Sleep, Sister Study (2003-2009), N = 38,007. Table S9. Adjusted [file 13098_2019_413_MOESM1_ESM.pdf]

Figure S1. Composition of Analytic Sample

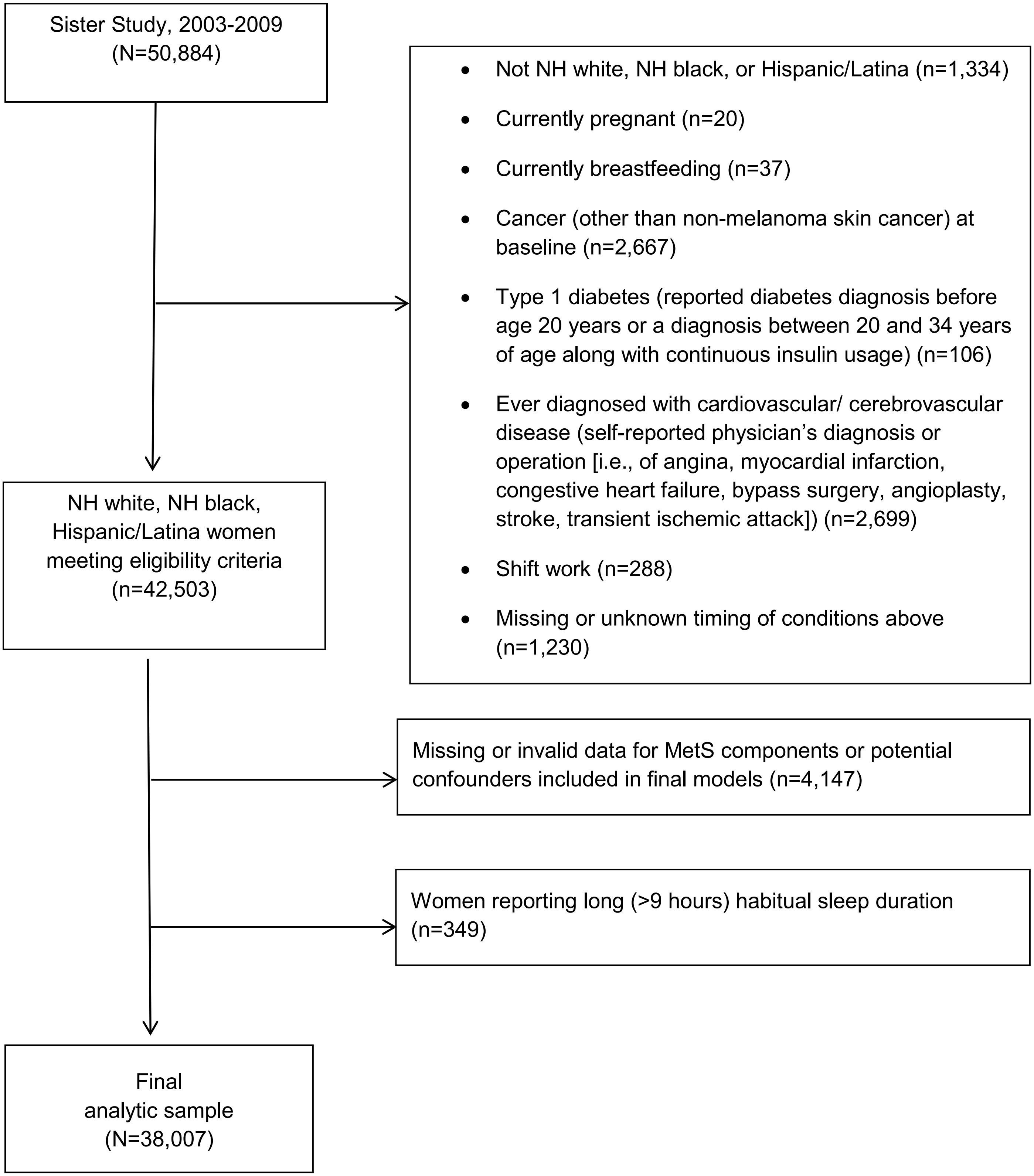

Table S1. Additionally Adjusted (for Use of Medication for Dyslipidemia) Prevalence Ratios for Metabolic Abnormalities Consistent with Metabolic Syndrome for Pre- and Post-Menopausal Women with Poor Sleep Compared to Women with Recommended Sleep, Sister Study (2003-2009), N=38,007

	**Total**	**White**	**Black**	**Hispanic**
	**Premenopausal Women**			
Sample size	N=13,988	n=11,757	n=1,417	n=814
n (%) with prevalent abnormalities consistent with MetS	787 (5.63)	541 (4.60)	175 (12.4)	71 (8.72)
	PR (95% CI)			
**Short sleep duration** (<7 h vs. recommended [7-9 h])	**1.25 (1.10-1.43)**	**1.25 (1.06-1.48)**	1.25 (0.97-1.61)	NE
**Inconsistent weekly sleep patterns** (yes vs. no)	1.07 (0.90-1.27)	1.10 (0.88-1.37)	0.99 (0.71-1.37)	NE
**Sleep debt** (yes vs. no) [B]	1.13 (0.97-1.31)	1.06 (0.87-1.29)	0.96 (0.71-1.30)	NE
**Napping ≥3 times/ week** (yes vs. no)	**1.25 (1.02-1.52)**	1.14 (0.88-1.49)	1.37 (0.97-1.94)	NE
**Insomnia symptoms** (yes vs. no)	1.15 (1.00-1.32)	1.11 (0.93-1.33)	1.10 (0.84-1.43)	NE
**Difficulty falling asleep** (yes vs. no)	1.11 (0.94-1.31)	1.11 (0.90-1.38)	1.08 (0.81-1.45)	NE
**Difficulty staying asleep** (yes vs. no)	**1.24 (1.04-1.46)**	1.17 (0.94-1.44)	1.26 (0.87-1.83)	NE
**Short sleep and insomnia symptoms** (yes vs. no)	**1.20 (1.01-1.42)**	1.18 (0.93-1.50)	1.11 (0.83-1.48)	NE
**Cumulative sleep score**	**1.09 (1.04-1.15)**	**1.08 (1.01-1.15)**	1.06 (0.97-1.17)	NE
	**Postmenopausal Women**			
Sample size	N=24,019	n=21,168	n=1,826	n=1,025
n (%) with prevalent abnormalities consistent with MetS	3,725 (15.5)	2,998 (14.2)	514 (28.2)	213 (20.8)
	PR (95% CI)			
**Short sleep duration** (<7 h vs. recommended [7-9 h])	**1.10 (1.04-1.17)**	**1.12 (1.05-1.19)**	1.05 (0.92-1.20)	1.20 (0.95-1.51)
**Inconsistent weekly sleep patterns** (yes vs. no)	**1.17 (1.10-1.25)**	**1.18 (1.10-1.27)**	**1.17 (1.02-1.35)**	1.13 (0.87-1.46)
**Sleep debt** (yes vs. no)	**1.21 (1.12-1.31)**	**1.22 (1.12-1.34)**	1.18 (1.00-1.39)	1.17 (0.89-1.52)
**Napping ≥3 times/ week** (yes vs. no)	**1.20 (1.12-1.29)**	**1.23 (1.13-1.33)**	**1.25 (1.08-1.45)**	1.02 (0.78-1.34)
**Insomnia symptoms** (yes vs. no)	**1.06 (1.00-1.13)**	**1.07 (1.00-1.14)**	1.01 (0.88-1.16)	1.10 (0.86-1.41)
**Difficulty falling asleep** (yes vs. no)	**1.08 (1.01-1.15)**	**1.08 (1.01-1.17)**	1.07 (0.93-1.24)	1.04 (0.81-1.32)
**Difficulty staying asleep** (yes vs. no) [A]	1.02 (0.95-1.09)	1.04 (0.96-1.13)	0.86 (0.71-1.04)	1.15 (0.87-1.50)
**Short sleep and insomnia symptoms** (yes vs. no) [A]	1.08 (1.00-1.16)	**1.13 (1.04-1.23)**	0.93 (0.79-1.10)	1.08 (0.83-1.41)
**Cumulative sleep score** [B]	**1.09 (1.06-1.11)**	**1.09 (1.07-1.12)**	**1.08 (1.03-1.13)**	**1.09 (0.99-1.19)**

$P_{\text{short sleep*menopausal status}}$ =0.0025; $P_{\text{consistent sleep*menopausal status}}$ =0.81; $P_{\text{sleep debt*menopausal status}}$ =0.43; $P_{\text{napping*menopausal status}}$ =0.22; $P_{\text{insomnia symptoms*menopausal status}}$ =0.011; $P_{\text{difficulty falling asleep*menopausal status}}$ =0.15; $P_{\text{difficulty staying asleep*menopausal status}}$ =0.0018; $P_{\text{short sleep and insomnia symptoms*menopausal status}}$ =0.0086; $P_{\text{sleep score *menopausal status}}$ =0.034

Abbreviations: MetS (metabolic syndrome); PR (prevalence ratio); CI (confidence interval); h (hours); NE (not estimable)

Adjusted for age at baseline (years), educational attainment (≤ high school graduate/graduation equivalent degree, some college/technical school/associate's degree, ≥ college graduate), annual household income (< $20,000, $20,000-$49,999, $50,000-$99,999, ≥ $100,000), diet quality (Healthy Eating Index score(38)), physical activity (METs [metabolic equivalent] hours per week), use of hormone replacement therapy (yes vs. no), alcohol consumption (nondrinker [former/never], light/moderate [≤7 drinks/week], heavy [>7 drinks/week]), smoking status (never, former, current), clinical depression or bipolar disorder (yes vs. no), and sleep medication use (yes vs. no). Models for sleep debt are also adjusted for consistent weekly sleep patterns (no vs. yes).
Bolded values indicate statistical significance at two-sided p=0.05.
[A] p <0.05 for interaction term (sleep variable by race/ethnicity)
[B] p <0.10 for interaction term (sleep variable by race/ethnicity)

Inconsistent weekly sleep patterns indicated whether participants reported consistent (could vary day-by-day but were stable from week-to-week) or inconsistent wake-up times and bedtimes during the prior six weeks. Sleep debt was defined as ≥2-hour difference between average longest and shortest sleep duration. Insomnia symptoms included difficulty falling asleep, defined as taking >30 minutes vs. ≤ 30 minutes to fall asleep on average, or difficulty staying asleep, defined as waking up ≥3 times per night ≥3 nights/week vs. <3 times per night <3 nights/week versus neither. Cumulative sleep score was the sum of yes responses to the main sleep characteristics (short sleep duration, inconsistent weekly sleep patterns, sleep debt, frequent napping, and insomnia symptoms [range: 0-5]).

<0.2% missingness for sleep medication use, habitual sleep duration category, consistent sleep pattern, sleep debt, napping, insomnia symptoms, difficulty falling asleep, difficulty staying asleep, cumulative sleep score

Table S2. Adjusted Prevalence Ratios of Metabolic Abnormalities Consistent with Metabolic Syndrome for Poor Sleep Compared to Recommended Sleep among Postmenopausal Women, Stratified by Type of Menopause, Sister Study (2003-2009), N=24,019

	Postmenopausal Women							
	**All (N=24,019)**		**White (n=21,168)**		**Black (n=1,826)**		**Hispanic (n=1,025)**	
**Natural Menopause**	Yes n=15,351 (63.9%)	No n=8,668 (36.1%)	Yes n=13,805 (65.2%)	No n=7,363 (34.8%)	Yes n=919 (50.3%)	No n=907 (49.7%)	Yes n=627 (61.2%)	No n=398 (38.8%)
n (%) with prevalent abnormalities consistent with MetS	2,030 (13.2)	1,695 (19.6)	1,676 (12.1)	1,322 (18.0)	239 (26.0)	275 (30.3)	115 (18.3)	98 (24.6)
	PR (95% CI)							
**Short sleep duration**	**1.12**	1.05	**1.16**	1.01	0.87	1.19	1.28	1.14
(<7 h vs. recommended [7-9 h])	**(1.03-1.23)**	(0.96-1.15)	**(1.05-1.27)**	(0.90-1.12)	(0.70-1.08)	(0.97-1.45)	(0.93-1.77)	(0.81-1.62)
**Inconsistent weekly sleep patterns**	**1.22**	**1.22**	**1.24**	**1.27**	1.10	1.09	1.22	1.02
(yes vs. no)	**(1.11-1.34)**	**(1.11-1.34)**	**(1.11-1.38)**	**(1.14-1.42)**	(0.86-1.41)	(0.88-1.34)	(0.85-1.75)	(0.69-1.51)
**Sleep debt**	**1.26**	**1.20**	**1.26**	**1.22**	1.08	1.23	**1.52**	0.88
(yes vs. no)	**(1.12-1.42)**	**(1.07-1.36)**	**(1.10-1.44)**	**(1.06-1.41)**	(0.81-1.43)	(0.96-1.57)	**(1.03-2.25)**	(0.57-1.34)
**Napping ≥3 times/ week**	**1.33**	**1.21**	**1.32**	**1.20**	**1.34**	**1.31**	1.23	1.03
(yes vs. no)	**(1.19-1.48)**	**(1.08-1.35)**	**(1.17-1.50)**	**(1.05-1.37)**	**(1.04-1.74)**	**(1.05-1.62)**	(0.83-1.82)	(0.69-1.55)
**Insomnia symptoms**	1.09	**1.12**	1.09	**1.13**	0.92	1.08	**1.45**	1.03
(yes vs. no)	(1.00-1.19)	**(1.02-1.22)**	(0.99-1.20)	**(1.02-1.25)**	(0.73-1.15)	(0.88-1.32)	**(1.03-2.03)**	(0.71-1.49)
**Difficulty falling asleep**	1.06	**1.15**	1.07	**1.18**	0.96	1.08	1.15	1.01
(yes vs. no)	(0.96-1.17)	**(1.04-1.26)**	(0.95-1.20)	**(1.05-1.31)**	(0.75-1.23)	(0.88-1.34)	(0.80-1.64)	(0.70-1.47)
**Difficulty staying asleep**	1.07	1.05	1.07	1.07	0.69	1.08	**2.04**	0.84
(yes vs. no)	(0.96-1.19)	(0.94-1.17)	(0.95-1.20)	(0.95-1.21)	(0.48-1.01)	(0.82-1.42)	**(1.46-2.87)**	(0.54-1.29)
**Short sleep and insomnia symptoms**	**1.15**	1.06	**1.20**	1.11	0.85	0.94	**1.46**	0.91
(yes vs. no)	**(1.03-1.29)**	(0.95-1.19)	**(1.06-1.37)**	(0.98-1.27)	(0.65-1.12)	(0.74-1.19)	**(1.01-2.10)**	(0.59-1.39)
**Cumulative sleep score**	**1.11**	**1.09**	**1.12**	**1.10**	1.02	**1.10**	**1.22**	1.03
	**(1.08-1.15)**	**(1.06-1.13)**	**(1.08-1.16)**	**(1.06-1.14)**	(0.93-1.11)	**(1.02-1.18)**	**(1.08-1.37)**	(0.90-1.17)

Abbreviations: MetS (metabolic syndrome); PR (prevalence ratio); CI (confidence interval); h (hours)

Adjusted for age at baseline (years), educational attainment (≤ high school graduate/graduation equivalent degree, some college/technical school/associate's degree, ≥ college graduate), annual household income (< $20,000, $20,000-$49,999, $50,000-$99,999, ≥ $100,000), diet quality (Healthy Eating Index score(38)), physical activity (METs [metabolic equivalent] hours per week),

use of hormone replacement therapy (yes vs. no), alcohol consumption (nondrinker [former/never], light/moderate [≤7 drinks/week], heavy [>7 drinks/week]), smoking status (never, former, current), clinical depression or bipolar disorder (yes vs. no), and sleep medication use (yes vs. no). Models for sleep debt are also adjusted for consistent weekly sleep patterns (no vs. yes).

Bolded values indicate statistical significance at two-sided p=0.05.

Note: Inconsistent weekly sleep patterns indicated whether participants reported consistent (could vary day-by-day but were stable from week-to-week) or inconsistent wake-up times and bedtimes during the prior six weeks. Sleep debt was defined as ≥2-hour difference between average longest and shortest sleep duration. Insomnia symptoms included difficulty falling asleep, defined as taking >30 minutes vs. ≤ 30 minutes to fall asleep on average, or difficulty staying asleep, defined as waking up ≥3 times per night ≥3 nights/week vs. <3 times per night <3 nights/week versus neither. Cumulative sleep score was the sum of yes responses to the main sleep characteristics (short sleep duration, inconsistent weekly sleep patterns, sleep debt, frequent napping, and insomnia symptoms [range: 0-5]).

<0.2% missingness for sleep medication use, habitual sleep duration category, consistent sleep pattern, sleep debt, napping, insomnia symptoms, difficulty falling asleep, difficulty staying asleep, cumulative sleep score

Table S3. Adjusted Prevalence Ratios of Metabolic Abnormalities Consistent with Metabolic Syndrome for Pre- and Post-Menopausal Women with Poor Sleep Compared to Women with Recommended Sleep using Elevated Diastolic Blood Pressure Cut Point of ≥80 mm Hg, Sister Study (2003-2009), N=38,007

	**Total**	**White**	**Black**	**Hispanic**
	**Premenopausal Women**			
Sample size	N=13,988	n=11,757	n=1,417	n=814
n (%) with prevalent abnormalities consistent with MetS	935 (6.68)	668 (5.68)	189 (13.3)	78 (9.58)
	PR (95% CI)			
**Short sleep duration** (<7 h vs. recommended [7-9 h])	**1.28 (1.12-1.46)**	**1.34 (1.15-1.57)**	1.17 (0.90-1.54)	NE
**Inconsistent weekly sleep patterns** (yes vs. no)	1.07 (0.90-1.27)	1.13 (0.92-1.38)	0.99 (0.71-1.39)	NE
**Sleep debt** (yes vs. no)	1.13 (0.98-1.31**)**	1.16 (0.97-1.38)	0.88 (0.65-1.20)	NE
**Napping ≥3 times/ week** (yes vs. no)	**1.26 (1.04-1.53)**	1.22 (0.96-1.55)	1.28 (0.88-1.86)	NE
**Insomnia symptoms** (yes vs. no)	**1.22 (1.06-1.39)**	1.12 (0.95-1.33)	1.32 (1.00-1.73)	NE
**Difficulty falling asleep** (yes vs. no)	1.13 (0.97-1.32)	1.10 (0.90-1.34)	1.14 (0.84-1.54)	NE
**Difficulty staying asleep** (yes vs. no)	**1.30 (1.10-1.53)**	1.18 (0.96-1.44)	**1.51 (1.07-2.14)**	NE
**Short sleep and insomnia symptoms** (yes vs. no)	**1.28 (1.08-1.52)**	**1.26 (1.01-1.57)**	1.28 (0.95-1.72)	NE
**Cumulative sleep score**	**1.11 (1.06-1.17)**	**1.11 (1.05-1.18)**	1.07 (0.96-1.19)	NE
	**Postmenopausal Women**			
Sample size	N=24,019	n=21,168	n=1,826	n=1,025
n (%) with prevalent abnormalities consistent with MetS	4,101 (17.1)	3,321 (15.7)	545 (29.9)	235 (22.9)
	PR (95% CI)			
**Short sleep duration** (<7 h vs. recommended [7-9 h])	**1.08 (1.02-1.15)**	**1.08 (1.01-1.16)**	1.07 (0.93-1.23)	1.18 (0.95-1.48)
**Inconsistent weekly sleep patterns** (yes vs. no)	**1.21 (1.13-1.29)**	**1.24 (1.15-1.33)**	1.11 (0.95-1.29)	1.18 (0.92-1.51)
**Sleep debt** (yes vs. no)	**1.22 (1.13-1.32)**	**1.23 (1.12-1.34)**	1.15 (0.96-1.37)	1.26 (0.96-1.65)
**Napping ≥3 times/ week** (yes vs. no)	**1.27 (1.18-1.36)**	**1.27 (1.17-1.39)**	**1.28 (1.09-1.51)**	1.17 (0.90-1.53)
**Insomnia symptoms** (yes vs. no) [A]	**1.13 (1.06-1.19)**	**1.14 (1.07-1.22)**	0.98 (0.85-1.14)	**1.31 (1.03-1.66)**
**Difficulty falling asleep** (yes)	**1.13 (1.06-1.21)**	**1.16 (1.07-1.25)**	1.01 (0.87-1.18)	1.14 (0.89-1.45)
**Difficulty staying asleep** (yes) [A]	1.07 (0.99-1.15)	1.08 (0.99-1.17)	0.88 (0.71-1.09)	**1.37 (1.07-1.77)**
**Short sleep and insomnia symptoms** (yes vs. no) [A]	**1.13 (1.05-1.22)**	**1.17 (1.08-1.28)**	0.92 (0.78-1.10)	1.26 (0.97-1.63)
**Cumulative sleep score** [A]	**1.10 (1.08-1.13)**	**1.11 (1.08-1.14)**	**1.06 (1.01-1.12)**	**1.15 (1.05-1.25)**

$P_{\text{short sleep*menopausal status}}$ =0.0011; $P_{\text{consistent sleep*menopausal status}}$ =0.99; $P_{\text{sleep debt*menopausal status}}$ =0.34; $P_{\text{napping*menopausal status}}$ =0.29; $P_{\text{insomnia symptoms*menopausal status}}$ =0.024; $P_{\text{difficulty falling asleep*menopausal status}}$ =0.23; $P_{\text{difficulty staying asleep*menopausal status}}$ =0.0049; $P_{\text{short sleep and insomnia symptoms*menopausal status}}$ =0.013; $P_{\text{sleep score *menopausal status}}$ =0.053

Abbreviations: MetS (metabolic syndrome); PR (prevalence ratio); CI (confidence interval); h (hours); NE (not estimable)

Adjusted for age at baseline (years), educational attainment (≤ high school graduate/graduation equivalent degree, some college/technical school/associate's degree, ≥ college graduate), annual household income (< $20,000, $20,000-$49,999, $50,000-$99,999, ≥ $100,000), diet quality (Healthy Eating Index score(38)), physical activity (METs [metabolic equivalent] hours per week), use of hormone replacement therapy (yes vs. no), alcohol consumption (nondrinker [former/never], light/moderate [≤7 drinks/week], heavy [>7 drinks/week]), smoking status (never, former, current), clinical depression or bipolar disorder (yes vs. no), and sleep medication use (yes vs. no). Models for sleep debt are also adjusted for consistent weekly sleep patterns (no vs. yes).

Bolded values indicate statistical significance at two-sided p=0.05.

[A] p <0.05 for interaction term (sleep variable by race/ethnicity)

[B] p <0.10 for interaction term (sleep variable by race/ethnicity)

Inconsistent weekly sleep patterns indicated whether participants reported consistent (could vary day-by-day but were stable from week-to-week) or inconsistent wake-up times and bedtimes during the prior six weeks. Sleep debt was defined as ≥2-hour difference between average longest and shortest sleep duration. Insomnia symptoms included difficulty falling asleep, defined as taking >30 minutes vs. ≤ 30 minutes to fall asleep on average, or difficulty staying asleep, defined as waking up ≥3 times per night ≥3 nights/week vs. <3 times per night <3 nights/week versus neither. Cumulative sleep score was the sum of yes responses to the main sleep characteristics (short sleep duration, inconsistent weekly sleep patterns, sleep debt, frequent napping, and insomnia symptoms [range: 0-5]).

<0.2% missingness for sleep medication use, habitual sleep duration category, consistent sleep pattern, sleep debt, napping, insomnia symptoms, difficulty falling asleep, difficulty staying asleep, cumulative sleep score

Table S4. Adjusted Prevalence Ratios of Metabolic Abnormalities Consistent with Metabolic Syndrome for Pre- and Post-Menopausal Women with Poor Sleep Compared to Women with Recommended Sleep Stratified by Sleep Medication Use, Sister Study (2003-2009), N=38,007

	Premenopausal Women							
	**All (N=13,988)**		**White (n=11,757)**		**Black (n=1,417)**		**Hispanic (n=814)**	
**Sleep medication use**	Yes n=2,782 (19.9%)	No n=11,206 (80.1%)	Yes n=2,403 (20.4%)	No n=9,354 (79.6%)	Yes n=216 (15.2%)	No n=1,201 (84.8%)	Yes n=163 (20.0%)	No n=651 (80.0%)
n (%) with prevalent abnormalities consistent with MetS	176 (6.33)	611 (5.45)	139 (5.78)	402 (4.30)	24 (11.1)	151 (12.6)	13 (7.98)	58 (8.91)
	PR (95% CI)							
**Short sleep duration** (<7 h vs. recommended [7-9 h])	1.00 (0.74-1.35)	**1.30 (1.11-1.54)**	1.00 (0.71-1.40)	**1.37 (1.12-1.68)**	0.96 (0.42-2.19)	1.27 (0.93-1.72)	NE	NE
**Inconsistent weekly sleep patterns** (yes vs. no)	1.08 (0.77-1.53)	1.04 (0.83-1.31)	1.28 (0.89-1.86)	1.06 (0.79-1.43)	0.39 (0.14-1.15)	1.14 (0.79-1.65)	NE	NE
**Sleep debt** (yes vs. no)	1.16 (0.81-1.66)	1.16 (0.97-1.38)	1.23 (0.81-1.87)	1.14 (0.91-1.43)	0.80 (0.28-2.32)	0.95 (0.68-1.31)	NE	NE
**Napping ≥3 times/ week** (yes vs. no)	0.95 (0.58-1.56)	**1.36 (1.07-1.72)**	0.95 (0.52-1.71)	1.27 (0.93-1.72)	0.95 (0.26-3.46)	1.42 (0.95-2.14)	NE	NE
**Insomnia symptoms** (yes vs. no)	1.03 (0.77-1.38)	**1.29 (1.09-1.52)**	1.11 (0.80-1.54)	1.08 (0.85-1.36)	0.76 (0.36-1.60)	**1.45 (1.07-1.95)**	NE	NE
**Difficulty falling asleep** (yes vs. no)	0.91 (0.66-1.26)	1.21 (0.99-1.48)	0.99 (0.68-1.43)	1.07 (0.79-1.43)	0.69 (0.34-1.41)	1.26 (0.90-1.76)	NE	NE
**Difficulty staying asleep** (yes vs. no)	1.30 (0.93-1.81)	**1.35 (1.10-1.67)**	1.37 (0.94-1.99)	1.13 (0.86-1.50)	0.82 (0.25-2.72)	**1.71 (1.17-2.50)**	NE	NE
**Short sleep and insomnia symptoms** (yes vs. no)	0.90 (0.63-1.29)	**1.42 (1.15-1.75)**	0.94 (0.62-1.44)	1.30 (0.95-1.77)	0.79 (0.34-1.83)	**1.43 (1.03-1.99)**	NE	NE
**Cumulative sleep score**	1.03 (0.93-1.15)	**1.13 (1.06-1.20)**	1.08 (0.95-1.22)	**1.11 (1.02-1.20)**	0.80 (0.61-1.05)	**1.14 (1.01-1.28)**	NE	NE
	**Postmenopausal Women**							
	**All (N=24,017)**		**White (n=21,166)**		**Black (n=1,826)**		**Hispanic (n=1,025)**	
**Sleep medication use**	Yes n=6,218 (25.9%)	No n=17,799 (74.1%)	Yes n=5,660 (26.7%)	No n=15,506 (73.3%)	Yes n=314 (17.2%)	No n=1,512 (82.8%)	Yes n=244 (23.8%)	No n=781 (76.2%)

n (%) with prevalent abnormalities consistent with MetS	1,048 (16.9)	2,677 (15.0)	904 (16.0)	2,094 (13.5)	88 (28.0)	426 (28.2)	56 (23.0)	157 (20.1)
	PR (95% CI)							
**Short sleep duration** (<7 h vs. recommended [7-9 h])	1.05 (0.93-1.18)	**1.10 (1.02-1.19)**	1.07 (0.94-1.21)	**1.09 (1.00-1.19)**	0.84 (0.58-1.20)	1.10 (0.94-1.29)	1.16 (0.74-1.83)	1.23 (0.93-1.62)
**Inconsistent weekly sleep patterns** (yes vs. no)	**1.27 (1.13-1.43)**	**1.20 (1.11-1.31)**	**1.34 (1.18-1.52)**	**1.22 (1.10-1.35)**	1.04 (0.72-1.50)	1.12 (0.94-1.34)	0.92 (0.55-1.55)	1.33 (0.98-1.81)
**Sleep debt** (yes vs. no)	**1.24 (1.06-1.46)**	**1.24 (1.12-1.37)**	**1.27 (1.06-1.51)**	**1.24 (1.10-1.40)**	1.13 (0.75-1.73)	1.18 (0.96-1.45)	1.19 (0.68-2.08)	1.22 (0.87-1.73)
**Napping ≥3 times/ week** (yes vs. no)	**1.26 (1.09-1.45)**	**1.27 (1.17-1.39)**	1.18 (0.99-1.40)	**1.30 (1.17-1.45)**	**1.63 (1.14-2.33)**	**1.29 (1.07-1.55)**	**1.94 (1.20-3.14)**	0.95 (0.65-1.37)
**Insomnia symptoms** (yes vs. no)	**1.18 (1.06-1.32)**	1.08 (1.00-1.17)	**1.19 (1.06-1.34)**	1.08 (0.99-1.18)	1.34 (0.93-1.93)	0.95 (0.80-1.13)	0.82 (0.50-1.33)	**1.39 (1.05-1.84)**
**Difficulty falling asleep** (yes vs. no)	**1.15 (1.02-1.28)**	1.09 (1.00-1.19)	**1.17 (1.04-1.33)**	1.10 (0.99-1.23)	1.32 (0.93-1.89)	0.97 (0.81-1.17)	0.68 (0.43-1.08)	1.29 (0.96-1.75)
**Difficulty staying asleep** (yes vs. no)	1.09 (0.96-1.24)	1.06 (0.96-1.17)	1.06 (0.92-1.22)	1.08 (0.97-1.20)	1.27 (0.86-1.87)	0.84 (0.65-1.09)	1.53 (0.96-2.44)	1.29 (0.92-1.81)
**Short sleep and insomnia symptoms** (yes vs. no)	1.12 (0.98-1.27)	**1.12 (1.01-1.23)**	1.15 (1.00-1.33)	**1.17 (1.04-1.33)**	1.01 (0.69-1.47)	0.92 (0.75-1.12)	1.01 (0.63-1.64)	1.24 (0.88-1.75)
**Cumulative sleep score**	**1.11 (1.07-1.16)**	**1.10 (1.07-1.14)**	**1.12 (1.07-1.17)**	**1.11 (1.07-1.14)**	1.07 (0.94-1.21)	**1.07 (1.01-1.14)**	1.07 (0.89-1.28)	**1.16 (1.04-1.29)**

Abbreviations: MetS (metabolic syndrome); PR (prevalence ratio); CI (confidence interval); h (hours); NE (not estimable)

Adjusted for age at baseline (years), educational attainment (≤ high school graduate/graduation equivalent degree, some college/technical school/associate's degree, ≥ college graduate), annual household income (< $20,000, $20,000-$49,999, $50,000-$99,999, ≥ $100,000), diet quality (Healthy Eating Index score(38)), physical activity (METs [metabolic equivalent] hours per week), use of hormone replacement therapy (yes vs. no), alcohol consumption (nondrinker [former/never], light/moderate [≤7 drinks/week], heavy [>7 drinks/week]), smoking status (never, former, current), and clinical depression or bipolar disorder (yes vs. no). Models for sleep debt are also adjusted for consistent weekly sleep patterns (no vs. yes).

Bolded values indicate statistical significance at two-sided p=0.05.

Inconsistent weekly sleep patterns indicated whether participants reported consistent (could vary day-by-day but were stable from week-to-week) or inconsistent wake-up times and bedtimes during the prior six weeks. Sleep debt was defined as ≥2-hour difference between average longest and shortest sleep duration. Insomnia symptoms included difficulty falling asleep, defined as taking >30 minutes vs. ≤ 30 minutes to fall asleep on average, or difficulty staying asleep, defined as waking up ≥3 times per night ≥3 nights/week vs. <3 times per night <3 nights/week versus neither. Cumulative sleep score was the sum of yes responses to the main sleep characteristics (short sleep duration, inconsistent weekly sleep patterns, sleep debt, frequent napping, and insomnia symptoms [range: 0-5]).

<0.2% missingness for sleep medication use, habitual sleep duration category, consistent sleep pattern, sleep debt, napping, insomnia symptoms, difficulty falling asleep, difficulty staying asleep, cumulative sleep score

Table S5. Additionally Adjusted (for other sleep characteristics) Prevalence Ratios of Metabolic Abnormalities Consistent with Metabolic Syndrome for Pre- and Post-Menopausal Women with Poor Sleep Compared to Women with Recommended Sleep, Sister Study (2003-2009), N=38,007

	**Premenopausal Women**			
	**Total (N=13,988)**	**White (n=11,757)**	**Black (n=1,417)**	**Hispanic (n=814)**
n (%) with prevalent abnormalities consistent with MetS	787 (5.63)	541 (4.60)	175 (12.4)	71 (8.72)
	PR (95% CI)			
**Short sleep duration** (<7 h vs. recommended [7-9 h])	**1.18 (1.02-1.38)**	**1.22 (1.01-1.47)**	1.15 (0.86-1.54)	NE
**Inconsistent weekly sleep patterns** (yes vs. no)	0.88 (0.71-1.09)	0.96 (0.73-1.26)	0.94 (0.64-1.38)	NE
**Sleep debt** (yes vs. no)	1.12 (0.96-1.32)	1.13 (0.92-1.38)	0.87 (0.64-1.19)	NE
**Napping ≥3 times/ week** (yes vs. no)	1.23 (0.99-1.52)	1.14 (0.87-1.50)	1.41 (0.96-2.08)	NE
**Insomnia symptoms** (yes vs. no)	**1.18 (1.01-1.37)**	1.04 (0.86-1.27)	1.31 (0.98-1.76)	NE
	**Postmenopausal Women**			
	**All (N=24,019)**	**White (n=21,168)**	**Black (n=1,826)**	**Hispanic (n=1,025)**
n (%) with prevalent abnormalities consistent with MetS	3,725 (15.5)	2,998 (14.2)	514 (28.2)	213 (20.8)
	PR (95% CI)			
**Short sleep duration** (<7 h vs. recommended [7-9 h])	1.03 (0.96-1.10)	1.02 (0.94-1.10)	1.04 (0.90-1.22)	1.17 (0.91-1.49)
**Inconsistent weekly sleep patterns** (yes vs. no)	1.03 (0.94-1.13)	1.05 (0.94-1.17)	0.99 (0.81-1.22)	0.98 (0.71-1.34)
**Sleep debt** (yes vs. no)	**1.23 (1.13-1.34)**	**1.24 (1.12-1.37)**	1.15 (0.95-1.38)	1.23 (0.92-1.63)
**Napping ≥3 times/ week** (yes vs. no)	**1.26 (1.17-1.36)**	**1.26 (1.15-1.37)**	**1.32 (1.11-1.56)**	1.17 (0.87-1.56)
**Insomnia symptoms** (yes vs. no) [B]	**1.08 (1.01-1.15)**	**1.08 (1.01-1.17)**	0.98 (0.84-1.14)	1.20 (0.93-1.55)

$P_{\text{short sleep*menopausal status}}$ =0.0097; $P_{\text{consistent sleep*menopausal status}}$ =0.68; $P_{\text{sleep debt*menopausal status}}$ =0.20; $P_{\text{napping*menopausal status}}$ =0.48; $P_{\text{insomnia symptoms*menopausal status}}$ =0.040

Abbreviations: MetS (metabolic syndrome); PR (prevalence ratio); CI (confidence interval); h (hours); NE (not estimable)

Adjusted for age at baseline (years), educational attainment (≤ high school graduate/graduation equivalent degree, some college/technical school/associate's degree, ≥ college graduate), annual household income (< $20,000, $20,000-$49,999, $50,000-$99,999, ≥ $100,000), diet quality (Healthy Eating Index score(38)), physical activity (METs [metabolic equivalent] hours per week), use of hormone replacement therapy (yes vs. no), alcohol consumption (nondrinker [former/never], light/moderate [≤7 drinks/week], heavy [>7 drinks/week]), smoking status (never, former, current), clinical depression or bipolar disorder (yes vs. no), sleep medication use (yes vs. no), and other sleep parameters. Therefore, models included short sleep duration, consistent weekly sleep patterns,

sleep debt, napping, and insomnia symptoms. Interaction terms for sleep parameters by menopausal status were estimated in separate models for each sleep parameter and not tested concurrently.

Inconsistent weekly sleep patterns indicated whether participants reported consistent (could vary day-by-day but were stable from week-to-week) or inconsistent wake-up times and bedtimes during the prior six weeks. Sleep debt was defined as ≥2-hour difference between average longest and shortest sleep duration. Insomnia symptoms included difficulty falling asleep, defined as taking >30 minutes vs. ≤ 30 minutes to fall asleep on average, or difficulty staying asleep, defined as waking up ≥3 times per night ≥3 nights/week vs. <3 times per night <3 nights/week versus neither.

Bolded values indicate statistical significance at two-sided p=0.05.

[A] p <0.05 for cross-product term (sleep variable by race/ethnicity)

[B] p <0.10 for cross-product term (sleep variable by race/ethnicity)

<0.2% missingness for sleep medication use, habitual sleep duration category, consistent sleep pattern, sleep debt, napping, insomnia symptoms

Table S6. Adjusted Prevalence Ratios of Hypertension for Pre- and Post-Menopausal Women with Poor Sleep Compared to Women with Recommended Sleep, Sister Study (2003-2009), N=38,007

	**Hypertension (original definition)** SSD, N, IS, DSA, CSS				**Hypertension (new guidelines)** SSD, CWSP, N, IS, DSA, SSD-IS, CSS			
	**Premenopausal Women**							
	**Total**	**White**	**Black**	**Hispanic**	**Total**	**White**	**Black**	**Hispanic**
Sample size	N=13,988	n=11,757	n=1,417	n=814	N=13,988	n=11,757	n=1,417	n=814
n (%) with prevalent hypertension	2,587 (18.5)	1,880 (16.0)	555 (39.2)	152 (18.7)	3,776 (27.0)	2,871 (24.4)	686 (48.4)	219 (26.9)
	PR (95% CI)				PR (95% CI)			
**Short sleep duration**	**1.10**	1.08	1.13	1.33	**1.10**	**1.12**	1.07	1.12
(<7 h vs. recommended [7-9 h])	**(1.03-1.19)**	(0.98-1.18)	(0.99-1.28)	(0.99-1.77)	**(1.04-1.17)**	**(1.04-1.20)**	(0.96-1.19)	(0.88-1.41)
**Inconsistent weekly sleep patterns**	1.03	1.01	1.10	1.07	**1.10**	1.10	**1.14**	1.08
(yes vs. no)	(0.94-1.14)	(0.89-1.15)	(0.94-1.28)	(0.74-1.56)	**(1.02-1.18)**	(1.00-1.20)	**(1.00-1.29)**	(0.81-1.44)
**Sleep debt**	**1.09**	1.10	1.05	1.04	**1.09**	**1.13**	0.98	0.98
(yes vs. no)	**(1.01-1.18)**	(1.00-1.22)	(0.91-1.22)	(0.75-1.46)	**(1.03-1.17)**	**(1.05-1.22)**	(0.87-1.12)	(0.74-1.30)
**Napping ≥3 times/ week**	**1.24**	**1.17**	**1.25**	**1.70**	**1.16**	**1.15**	1.06	**1.53**
(yes vs. no)	**(1.11-1.38)**	**(1.02-1.35)**	**(1.05-1.50)**	**(1.18-2.46)**	**(1.06-1.26)**	**(1.03-1.29)**	(0.90-1.25)	**(1.15-2.06)**
**Insomnia symptoms**	**1.09**	1.10	1.06	1.24	**1.10**	**1.10**	1.08	1.19
(yes vs. no)	**(1.01-1.18)**	(0.99-1.21)	(0.92-1.21)	(0.92-1.68)	**(1.04-1.17)**	**(1.03-1.19)**	(0.97-1.21)	(0.93-1.52)
**Difficulty falling asleep**	1.04	1.07	1.00	1.05	**1.08**	1.10	1.04	1.07
(yes vs. no)	(0.95-1.14)	(0.95-1.20)	(0.86-1.16)	(0.75-1.46)	**(1.01-1.16)**	(1.01-1.20)	(0.92-1.17)	(0.82-1.40)
**Difficulty staying asleep**	**1.16**	**1.15**	**1.20**	1.12	**1.13**	**1.12**	**1.20**	1.07
(yes vs. no)	**(1.05-1.27)**	**(1.02-1.29)**	**(1.01-1.43)**	(0.75-1.66)	**(1.05-1.22)**	**(1.02-1.22)**	**(1.03-1.38)**	(0.77-1.47)
**Short sleep and insomnia symptoms**	1.04	1.05	1.01	1.23	**1.08**	**1.13**	1.01	1.11
(yes vs. no)	(0.94-1.15)	(0.92-1.20)	(0.87-1.18)	(0.85-1.77)	**(1.00-1.17)**	**(1.02-1.25)**	(0.89-1.15)	(0.82-1.51)
**Cumulative sleep score**	**1.06**	**1.05**	**1.06**	**1.14**	**1.07**	**1.07**	**1.04**	1.09
	**(1.03-1.09)**	**(1.01-1.09)**	**(1.01-1.12)**	**(1.02-1.28)**	**(1.04-1.09)**	**(1.04-1.10)**	**(1.00-1.09)**	(1.00-1.19)
	**Postmenopausal Women**							
	**Total**	**White**	**Black**	**Hispanic**	**Total**	**White**	**Black**	**Hispanic**
Sample size	N=24,019	n=21,168	n=1,826	n=1,025	N=24,019	n=21,168	n=1,826	n=1,025
n (%) with prevalent hypertension	8,933 (37.2)	7,490 (35.4)	1,050 (57.5)	393 (38.3)	10,832 (45.1)	9,169 (43.3)	1,187 (65.0)	476 (46.4)
	PR (95% CI)				PR (95% CI)			
**Short sleep duration**	1.02	1.03	0.94	1.07	1.01	1.01	0.96	1.06
(<7 h vs. recommended [7-9 h])	(0.98-1.05)	(0.99-1.07)	(0.87-1.01)	(0.92-1.25)	(0.98-1.04)	(0.98-1.05)	(0.89-1.02)	(0.93-1.21)
**Inconsistent weekly sleep patterns**	**1.05**	**1.05**	1.03	1.07	1.04	1.04	1.04	1.03

(yes vs. no)	**(1.01-1.09)**	**(1.01-1.10)**	(0.94-1.13)	(0.89-1.27)	(1.00-1.07)	(1.00-1.08)	(0.97-1.13)	(0.88-1.20)
**Sleep debt**	**1.09** [A]	**1.13**	0.94	0.95	**1.08** [A]	**1.11**	0.94	1.04
(yes vs. no)	**(1.04-1.15)**	**(1.07-1.20)**	(0.85-1.05)	(0.77-1.17)	**(1.03-1.12)**	**(1.06-1.16)**	(0.86-1.03)	(0.88-1.24)
**Napping ≥3 times/ week**	**1.10**	**1.11**	1.10	1.02	**1.09**	**1.09**	1.08	1.08
(yes vs. no)	**(1.05-1.15)**	**(1.05-1.17)**	(1.00-1.21)	(0.84-1.24)	**(1.05-1.13)**	**(1.05-1.14)**	(0.99-1.17)	(0.91-1.27)
**Insomnia symptoms**	**1.06**	**1.07**	1.05	1.05	**1.06**	**1.07**	1.03	1.06
(yes vs. no)	**(1.03-1.10)**	**(1.03-1.11)**	(0.97-1.14)	(0.90-1.24)	**(1.03-1.09)**	**(1.03-1.10)**	(0.96-1.10)	(0.92-1.22)
**Difficulty falling asleep**	**1.06**	**1.06**	1.05	1.01	**1.06**	**1.06**	1.06	1.00
(yes vs. no)	**(1.02-1.10)**	**(1.01-1.11)**	(0.97-1.15)	(0.86-1.20)	**(1.02-1.09)**	**(1.02-1.10)**	(0.98-1.14)	(0.86-1.15)
**Difficulty staying asleep**	**1.05**	**1.05**	1.05	1.11	**1.05**	**1.05**	0.99	1.12
(yes vs. no)	**(1.01-1.10)**	**(1.00-1.10)**	(0.93-1.18)	(0.92-1.34)	**(1.01-1.09)**	**(1.01-1.09)**	(0.90-1.10)	(0.96-1.32)
**Short sleep and insomnia symptoms**	**1.05**	**1.07**	0.99	0.99	**1.06** [B]	**1.07**	0.99	1.08
(yes vs. no)	**(1.00-1.10)**	**(1.02-1.13)**	(0.90-1.09)	(0.82-1.21)	**(1.02-1.10)**	**(1.02-1.12)**	(0.91-1.07)	(0.92-1.26)
**Cumulative sleep score**	**1.04** [A]	**1.04**	1.00	1.03	**1.03** [A]	**1.03**	1.00	1.03
	**(1.02-1.05)**	**(1.03-1.06)**	(0.97-1.03)	(0.97-1.10)	**(1.02-1.04)**	**(1.02-1.05)**	(0.98-1.03)	(0.98-1.09)

[SSD] $P_{\text{short sleep duration*menopausal status}}<0.05$; [CWSP] $P_{\text{consistent weekly sleep paptters*menopausal status}}<0.05$; [N] $P_{\text{napping*menopausal status}}<0.05$; [IS] $P_{\text{insomnia symptoms*menopausal status}}<0.05$; [DSA] $P_{\text{difficulty staying asleep*menopausal status}}<0.05$; [SSD-IS] $P_{\text{short sleep duration and insomnia symptoms*menopausal status}}<0.05$; [CSS] $P_{\text{cumulative sleep score*menopausal status}}<0.05$

Abbreviations: PR (prevalence ratio); CI (confidence interval); h (hours)

Adjusted for age at baseline (years), educational attainment (≤ high school graduate/graduation equivalent degree, some college/technical school/associate's degree, ≥ college graduate), annual household income (< $20,000, $20,000-$49,999, $50,000-$99,999, ≥ $100,000), diet quality (Healthy Eating Index score(38)), physical activity (METs [metabolic equivalent] hours per week), use of hormone replacement therapy (yes vs. no), alcohol consumption (nondrinker [former/never], light/moderate [≤7 drinks/week], heavy [>7 drinks/week]), smoking status (never, former, current), clinical depression or bipolar disorder (yes vs. no), and sleep medication use (yes vs. no). Models for sleep debt are also adjusted for consistent weekly sleep patterns (no vs. yes).

Bolded values indicate statistical significance at two-sided p=0.05.

[A] p <0.05 for interaction term (sleep variable by race/ethnicity)

[B] p <0.10 for interaction term (sleep variable by race/ethnicity)

Original definition (systolic blood pressure (blood pressure) > 130 mm Hg or diastolic BP > 85 mm Hg) as defined by Alberti et al. (2009) and new guidelines refer to the statement of the American College of Cardiology/American Heart Association Task Force (2017) (systolic BP > 130 mm Hg or diastolic BP > 80 mm Hg).

Inconsistent weekly sleep patterns indicated whether participants reported consistent (could vary day-by-day but were stable from week-to-week) or inconsistent wake-up times and bedtimes during the prior six weeks. Sleep debt was defined as ≥2-hour difference between average longest and shortest sleep duration. Insomnia symptoms included difficulty falling asleep, defined as taking >30 minutes vs. ≤ 30 minutes to fall asleep on average, or difficulty staying asleep, defined as waking up ≥3 times per night ≥3 nights/week vs. <3 times per night <3 nights/week versus neither. Cumulative sleep score was the sum of yes responses to the main sleep characteristics (short sleep duration, inconsistent weekly sleep patterns, sleep debt, frequent napping, and insomnia symptoms [range: 0-5]).

<0.2% missingness for sleep medication use, habitual sleep duration category, consistent sleep pattern, sleep debt, napping, insomnia symptoms, difficulty falling asleep, difficulty staying asleep, cumulative sleep score

Table S7. Adjusted Prevalence Ratios of Abdominal Obesity for Pre- and Post-Menopausal Women with Poor Sleep Compared to Women with Recommended Sleep, Sister Study (2003-2009), N=38,007

	Total	White	Black	Hispanic
	**Premenopausal Women**			
Sample size	N=13,988	n=11,757	n=1,417	n=814
n (%) with prevalent abdominal obesity	4,890 (35.0)	3,803 (32.4)	770 (54.3)	317 (38.9)
	PR (95% CI)			
**Short sleep duration** (<7 h vs. recommended [7-9 h]) [A]	**1.15 (1.09-1.20)**	**1.18 (1.12-1.25)**	1.01 (0.92-1.11)	1.12 (0.95-1.34)
**Inconsistent weekly sleep patterns** (yes vs. no) [B]	**1.11 (1.04-1.17)**	**1.13 (1.05-1.21)**	1.05 (0.93-1.17)	1.17 (0.95-1.44)
**Sleep debt** (yes vs. no)	**1.12 (1.06-1.18)**	**1.12 (1.06-1.20)**	1.09 (0.98-1.22)	1.04 (0.86-1.27)
**Napping ≥3 times/ week** (yes vs. no)	**1.15 (1.07-1.23)**	**1.16 (1.06-1.26)**	1.12 (0.97-1.28)	1.12 (0.87-1.45)
**Insomnia symptoms** (yes vs. no) [A]	**1.11 (1.06-1.17)**	**1.15 (1.08-1.22)**	1.05 (0.95-1.16)	0.98 (0.81-1.18)
**Difficulty falling asleep** (yes vs. no) [A]	**1.11 (1.05-1.18)**	**1.15 (1.07-1.23)**	1.07 (0.96-1.19)	0.97 (0.80-1.19)
**Difficulty staying asleep** (yes vs. no) [A]	**1.10 (1.03-1.17)**	**1.13 (1.05-1.22)**	1.01 (0.87-1.18)	0.91 (0.70-1.19)
**Short sleep and insomnia symptoms** (yes vs. no) [A]	**1.11 (1.04-1.19)**	**1.15 (1.06-1.25)**	1.04 (0.93-1.16)	1.10 (0.89-1.37)
**Cumulative sleep score** [A]	**1.08 (1.06-1.10)**	**1.09 (1.07-1.11)**	1.03 (0.99-1.07)	1.05 (0.98-1.13)
	**Postmenopausal Women**			
Sample size	N=24,019	n=21,168	n=1,826	n=1,025
n (%) with prevalent abdominal obesity	10,087 (42.0)	8,451 (39.9)	1,148 (62.9)	488 (47.6)
	PR (95% CI)			
**Short sleep duration** (<7 h vs. recommended [7-9 h]) [A]	**1.05 (1.02-1.08)**	**1.04 (1.01-1.08)**	1.02 (0.95-1.09)	**1.26 (1.11-1.43)**
**Inconsistent weekly sleep patterns** (yes vs. no) [A]	**1.14 (1.10-1.18)**	**1.15 (1.11-1.20)**	1.06 (0.99-1.15)	**1.20 (1.04-1.37)**
**Sleep debt** (yes vs. no)	**1.13 (1.08-1.17)**	**1.13 (1.08-1.18)**	**1.14 (1.04-1.24)**	1.10 (0.94-1.29)
**Napping ≥3 times/ week** (yes vs. no) [A]	**1.17 (1.12-1.21)**	**1.19 (1.14-1.24)**	**1.12 (1.03-1.21)**	1.05 (0.89-1.24)
**Insomnia symptoms** (yes vs. no)	**1.06 (1.03-1.09)**	**1.06 (1.03-1.10)**	1.02 (0.95-1.10)	1.12 (0.98-1.27)
**Difficulty falling asleep** (yes vs. no) [B]	**1.07 (1.03-1.11)**	**1.08 (1.04-1.13)**	1.02 (0.95-1.10)	1.04 (0.90-1.19)
**Difficulty staying asleep** (yes vs. no) [A]	1.03 (0.99-1.07)	1.03 (0.98-1.07)	0.99 (0.89-1.09)	**1.24 (1.08-1.44)**
**Short sleep and insomnia symptoms** (yes vs. no) [A]	**1.05 (1.01-1.09)**	**1.06 (1.01-1.11)**	0.96 (0.89-1.05)	**1.23 (1.07-1.42)**
**Cumulative sleep score** [A]	**1.06 (1.05-1.08)**	**1.07 (1.05-1.08)**	**1.04 (1.01-1.06)**	**1.11 (1.05-1.16)**

$P_{\text{short sleep*menopausal status}}$ =0.00020; $P_{\text{consistent sleep*menopausal status}}$ =0.94; $P_{\text{sleep debt*menopausal status}}$ =0.69; $P_{\text{napping*menopausal status}}$ =0.64; $P_{\text{insomnia symptoms*menopausal status}}$ =0.018; $P_{\text{difficulty falling asleep*menopausal status}}$ =0.067; $P_{\text{difficulty staying asleep*menopausal status}}$ =0.036; $P_{\text{short sleep and insomnia symptoms*menopausal status}}$ =0.029; $P_{\text{sleep score *menopausal status}}$ =0.029

Abbreviations: MetS (metabolic syndrome); PR (prevalence ratio); CI (confidence interval); h (hours)

Adjusted for age at baseline (years), educational attainment (≤ high school graduate/graduation equivalent degree, some college/technical school/associate’s degree, ≥ college graduate), annual household income (< $20,000, $20,000-$49,999, $50,000-$99,999, ≥ $100,000), diet quality (Healthy Eating Index score(38)), physical activity (METs [metabolic equivalent] hours per week), use of hormone replacement therapy (yes vs. no), alcohol consumption (nondrinker [former/never], light/moderate [≤7 drinks/week], heavy [>7 drinks/week]), smoking status (never, former, current), clinical depression or bipolar disorder (yes vs. no), and sleep medication use (yes vs. no). Models for sleep debt are also adjusted for consistent weekly sleep patterns (no vs. yes).

Bolded values indicate statistical significance at two-sided p=0.05.

[A] p <0.05 for interaction term (sleep variable by race/ethnicity)

[B] p <0.10 for interaction term (sleep variable by race/ethnicity)

Inconsistent weekly sleep patterns indicated whether participants reported consistent (could vary day-by-day but were stable from week-to-week) or inconsistent wake-up times and bedtimes during the prior six weeks. Sleep debt was defined as ≥2-hour difference between average longest and shortest sleep duration. Insomnia symptoms included difficulty falling asleep, defined as taking >30 minutes vs. ≤ 30 minutes to fall asleep on average, or difficulty staying asleep, defined as waking up ≥3 times per night ≥3 nights/week vs. <3 times per night <3 nights/week versus neither. Cumulative sleep score was the sum of yes responses to the main sleep characteristics (short sleep duration, inconsistent weekly sleep patterns, sleep debt, frequent napping, and insomnia symptoms [range: 0-5]).

<0.2% missingness for sleep medication use, habitual sleep duration category, consistent sleep pattern, sleep debt, napping, insomnia symptoms, difficulty falling asleep, difficulty staying asleep, cumulative sleep score

Table S8. Adjusted Prevalence Ratios of Dyslipidemia for Pre- and Post-Menopausal Women with Poor Sleep Compared to Women with Recommended Sleep, Sister Study (2003-2009), N=38,007

	**Total**	**White**	**Black**	**Hispanic**
	**Premenopausal Women**			
Sample size	N=13,988	n=11,757	n=1,417	n=814
n (%) with prevalent dyslipidemia	2,692 (19.3)	2,166 (18.4)	325 (22.9)	201 (24.7)
	PR (95% CI)			
**Short sleep duration** (<7 h vs. recommended [7-9 h])	1.04 (0.96-1.12)	1.04 (0.96-1.14)	1.02 (0.85-1.24)	1.03 (0.81-1.32)
**Inconsistent weekly sleep patterns** (yes vs. no)	1.03 (0.93-1.14)	1.05 (0.94-1.18)	0.92 (0.71-1.19)	1.05 (0.78-1.42)
**Sleep debt** (yes vs. no)	1.03 (0.95-1.12)	1.05 (0.96-1.16)	0.95 (0.76-1.19)	0.94 (0.70-1.24)
**Napping ≥3 times/ week** (yes vs. no)	1.10 (0.98-1.23)	1.08 (0.94-1.24)	1.00 (0.74-1.35)	1.37 (0.99-1.89)
**Insomnia symptoms** (yes vs. no)	**1.13 (1.05-1.23)**	**1.12 (1.03-1.23)**	1.19 (0.97-1.45)	1.17 (0.91-1.51)
**Difficulty falling asleep** (yes vs. no)	1.08 (0.99-1.18)	1.08 (0.97-1.20)	1.05 (0.84-1.31)	1.20 (0.93-1.56)
**Difficulty staying asleep** (yes vs. no)	**1.17 (1.07-1.29)**	**1.15 (1.03-1.28)**	**1.36 (1.04-1.77)**	1.17 (0.86-1.60)
**Short sleep and insomnia symptoms** (yes vs. no)	**1.13 (1.02-1.25)**	**1.15 (1.01-1.29)**	1.09 (0.87-1.37)	1.23 (0.91-1.67)
**Cumulative sleep score**	**1.04 (1.01-1.07)**	**1.04 (1.01-1.08)**	1.01 (0.93-1.09)	1.06 (0.96-1.17)
	**Postmenopausal Women**			
Sample size	N=24,019	n=21,168	n=1,826	n=1,025
n (%) with prevalent dyslipidemia	9,768 (40.7)	8,487 (40.1)	770 (42.2)	511 (49.9)
	PR (95% CI)			
**Short sleep duration** (<7 h vs. recommended [7-9 h])	1.00 (0.96-1.03)	0.99 (0.95-1.03)	1.07 (0.96-1.19)	1.03 (0.91-1.16)
**Inconsistent weekly sleep patterns** (yes vs. no)	**1.07 (1.03-1.11)**	**1.08 (1.04-1.13)**	0.97 (0.86-1.10)	1.03 (0.90-1.19)
**Sleep debt** (yes vs. no)	1.05 (1.00-1.10)	1.05 (1.00-1.10)	0.96 (0.83-1.12)	1.13 (0.97-1.32)
**Napping ≥3 times/ week** (yes vs. no)	**1.07 (1.02-1.12)**	**1.07 (1.01-1.12)**	1.14 (0.99-1.30)	1.04 (0.89-1.21)
**Insomnia symptoms** (yes vs. no)	**1.09 (1.05-1.12)**	**1.09 (1.06-1.13)**	1.00 (0.90-1.13)	1.14 (1.00-1.29)
**Difficulty falling asleep** (yes vs. no) [B]	**1.07 (1.03-1.11)**	**1.09 (1.04-1.13)**	0.95 (0.84-1.08)	1.10 (0.97-1.25)
**Difficulty staying asleep** (yes vs. no)	**1.06 (1.02-1.10)**	**1.06 (1.02-1.11)**	1.03 (0.88-1.20)	1.08 (0.93-1.25)
**Short sleep and insomnia symptoms** (yes vs. no)	**1.06 (1.01-1.10)**	**1.06 (1.01-1.12)**	0.98 (0.86-1.12)	1.12 (0.97-1.29)
**Cumulative sleep score**	**1.03 (1.02-1.05)**	**1.03 (1.02-1.05)**	1.01 (0.97-1.06)	1.05 (1.00-1.10)

$P_{\text{short sleep*menopausal status}}$ =0.049; $P_{\text{consistent sleep*menopausal status}}$ =0.59; $P_{\text{sleep debt*menopausal status}}$ =0.86; $P_{\text{napping*menopausal status}}$ =0.19; $P_{\text{insomnia symptoms*menopausal status}}$ =0.020; $P_{\text{difficulty falling asleep*menopausal status}}$ =0.12; $P_{\text{difficulty staying asleep*menopausal status}}$ =0.0095; $P_{\text{short sleep and insomnia symptoms*menopausal status}}$ =0.019; $P_{\text{sleep score *menopausal status}}$ =0.031

Abbreviations: MetS (metabolic syndrome); PR (prevalence ratio); CI (confidence interval); h (hours)

Adjusted for age at baseline (years), educational attainment (≤ high school graduate/graduation equivalent degree, some college/technical school/associate's degree, ≥ college graduate), annual household income (< $20,000, $20,000-$49,999, $50,000-$99,999, ≥ $100,000), diet quality (Healthy Eating Index score(38)), physical activity (METs [metabolic equivalent] hours per week), use of hormone replacement therapy (yes vs. no), alcohol consumption (nondrinker [former/never], light/moderate [≤7 drinks/week], heavy [>7 drinks/week]), smoking status (never, former, current), clinical depression or bipolar disorder (yes vs. no), and sleep medication use (yes vs. no). Models for sleep debt are also adjusted for consistent weekly sleep patterns (no vs. yes).

Bolded values indicate statistical significance at two-sided p=0.05.

[A] p <0.05 for interaction term (sleep variable by race/ethnicity)

[B] p <0.10 for interaction term (sleep variable by race/ethnicity)

Inconsistent weekly sleep patterns indicated whether participants reported consistent (could vary day-by-day but were stable from week-to-week) or inconsistent wake-up times and bedtimes during the prior six weeks. Sleep debt was defined as ≥2-hour difference between average longest and shortest sleep duration. Insomnia symptoms included difficulty falling asleep, defined as taking >30 minutes vs. ≤ 30 minutes to fall asleep on average, or difficulty staying asleep, defined as waking up ≥3 times per night ≥3 nights/week vs. <3 times per night <3 nights/week versus neither. Cumulative sleep score was the sum of yes responses to the main sleep characteristics (short sleep duration, inconsistent weekly sleep patterns, sleep debt, frequent napping, and insomnia symptoms [range: 0-5]).

<0.2% missingness for sleep medication use, habitual sleep duration category, consistent sleep pattern, sleep debt, napping, insomnia symptoms, difficulty falling asleep, difficulty staying asleep, cumulative sleep score

Table S9. Adjusted Prevalence Ratios of Prediabetes/Type 2 Diabetes Mellitus (T2DM) for Pre- and Post-Menopausal Women with Poor Sleep Compared to Women with Recommended Sleep, Sister Study (2003-2009), N=38,007

	**Total**	**White**	**Black**	**Hispanic**
	**Premenopausal Women**			
Sample size	N=13,988	n=11,757	n=1,417	n=814
n (%) with prevalent prediabetes/T2DM	599 (4.28)	408 (3.47)	125 (8.82)	66 (8.11)
	PR (95% CI)			
**Short sleep duration** (<7 h vs. recommended [7-9 h])	**1.20 (1.01-1.43)**	**1.35 (1.10-1.66)**	0.97 (0.69-1.36)	NE
**Inconsistent weekly sleep patterns** (yes vs. no)	1.03 (0.83-1.28)	1.05 (0.80-1.38)	1.07 (0.70-1.63)	NE
**Sleep debt** (yes vs. no)	1.14 (0.95-1.37)	1.13 (0.89-1.42)	0.96 (0.66-1.40)	NE
**Napping ≥3 times/ week** (yes vs. no)	**1.35 (1.06-1.72)**	1.32 (0.97-1.79)	1.21 (0.73-1.98)	NE
**Insomnia symptoms** (yes vs. no)	1.13 (0.95-1.35)	1.01 (0.81-1.27)	1.20 (0.83-1.72)	NE
**Difficulty falling asleep** (yes vs. no)	1.00 (0.82-1.23)	0.88 (0.66-1.15)	1.04 (0.69-1.56)	NE
**Difficulty staying asleep** (yes vs. no)	**1.34 (1.08-1.65)**	1.27 (0.98-1.65)	1.42 (0.90-2.24)	NE
**Short sleep and insomnia symptoms** (yes vs. no)	**1.26 (1.01-1.56)**	1.18 (0.87-1.59)	1.32 (0.90-1.94)	NE
**Cumulative sleep score**	**1.09 (1.02-1.17)**	**1.09 (1.01-1.18)**	1.04 (0.91-1.20)	NE
	**Postmenopausal Women**			
Sample size	N=24,019	n=21,168	n=1,826	n=1,025
n (%) with prevalent prediabetes/T2DM	2,057 (8.56)	1,566 (7.40)	337 (18.5)	154 (15.0)
	PR (95% CI)			
**Short sleep duration** (<7 h vs. recommended [7-9 h])	1.09 (1.00-1.19)	1.10 (1.00-1.22)	1.06 (0.87-1.29)	1.13 (0.85-1.52)
**Inconsistent weekly sleep patterns** (yes vs. no)	**1.28 (1.16-1.41)**	**1.33 (1.19-1.49)**	1.18 (0.96-1.46)	1.12 (0.81-1.56)
**Sleep debt** (yes vs. no)	**1.15 (1.02-1.29)**	1.10 (0.95-1.28)	1.08 (0.84-1.40)	**1.62 (1.13-2.33)**
**Napping ≥3 times/ week** (yes vs. no)	**1.40 (1.26-1.56)**	**1.40 (1.23-1.59)**	**1.42 (1.13-1.77)**	**1.45 (1.05-2.02)**
**Insomnia symptoms** (yes vs. no)	**1.12 (1.03-1.23)**	**1.12 (1.01-1.24)**	1.11 (0.91-1.35)	1.25 (0.92-1.70)
**Difficulty falling asleep** (yes vs. no)	**1.14 (1.03-1.26)**	**1.14 (1.01-1.28)**	1.17 (0.95-1.43)	1.12 (0.81-1.54)
**Difficulty staying asleep** (yes vs. no) [B]	1.09 (0.98-1.21)	1.12 (0.99-1.26)	0.87 (0.65-1.16)	1.32 (0.95-1.85)
**Short sleep and insomnia symptoms** (yes vs. no)	1.10 (0.98-1.22)	1.11 (0.96-1.27)	1.04 (0.83-1.30)	1.28 (0.92-1.78)
**Cumulative sleep score**	**1.12 (1.09-1.16)**	**1.12 (1.08-1.17)**	**1.10 (1.03-1.18)**	**1.17 (1.05-1.31)**

$P_{\text{short sleep*menopausal status}}$ =0.20; $P_{\text{consistent sleep*menopausal status}}$ =0.22; $P_{\text{sleep debt*menopausal status}}$ =0.43; $P_{\text{napping*menopausal status}}$ =0.77; $P_{\text{insomnia symptoms*menopausal status}}$ =0.56; $P_{\text{difficulty falling asleep*menopausal status}}$ =0.52; $P_{\text{difficulty staying asleep*menopausal status}}$ =0.045; $P_{\text{short sleep and insomnia symptoms*menopausal status}}$ =0.15; $P_{\text{sleep score *menopausal status}}$ =0.96

Abbreviations: MetS (metabolic syndrome); PR (prevalence ratio); CI (confidence interval); h (hours); NE (not estimable)

Adjusted for age at baseline (years), educational attainment (≤ high school graduate/graduation equivalent degree, some college/technical school/associate's degree, ≥ college graduate), annual household income (< $20,000, $20,000-$49,999, $50,000-$99,999, ≥ $100,000), diet quality (Healthy Eating Index score(38)), physical activity (METs [metabolic equivalent] hours per week), use of hormone replacement therapy (yes vs. no), alcohol consumption (nondrinker [former/never], light/moderate [≤7 drinks/week], heavy [>7 drinks/week]), smoking status (never, former, current), clinical depression or bipolar disorder (yes vs. no), and sleep medication use (yes vs. no). Models for sleep debt are also adjusted for consistent weekly sleep patterns (no vs. yes).

Bolded values indicate statistical significance at two-sided p=0.05.

[A] p <0.05 for interaction term (sleep variable by race/ethnicity)

[B] p <0.10 for interaction term (sleep variable by race/ethnicity)

Inconsistent weekly sleep patterns indicated whether participants reported consistent (could vary day-by-day but were stable from week-to-week) or inconsistent wake-up times and bedtimes during the prior six weeks. Sleep debt was defined as ≥2-hour difference between average longest and shortest sleep duration. Insomnia symptoms included difficulty falling asleep, defined as taking >30 minutes vs. ≤ 30 minutes to fall asleep on average, or difficulty staying asleep, defined as waking up ≥3 times per night ≥3 nights/week vs. <3 times per night <3 nights/week versus neither. Cumulative sleep score was the sum of yes responses to the main sleep characteristics (short sleep duration, inconsistent weekly sleep patterns, sleep debt, frequent napping, and insomnia symptoms [range: 0-5]).

<0.2% missingness for sleep medication use, habitual sleep duration category, consistent sleep pattern, sleep debt, napping, insomnia symptoms, difficulty falling asleep, difficulty staying asleep, cumulative sleep score
